# Prevention and Control of Seasonal Influenza with Vaccines: Recommendations of the Advisory Committee on Immunization Practices — United States, 2022–23 Influenza Season

**DOI:** 10.15585/mmwr.rr7101a1

**Published:** 2022-08-26

**Authors:** Lisa A. Grohskopf, Lenee H. Blanton, Jill M. Ferdinands, Jessie R. Chung, Karen R. Broder, H. Keipp Talbot, Rebecca L. Morgan, Alicia M. Fry

**Affiliations:** ^1^Influenza Division, National Center for Immunization and Respiratory Diseases, CDC; ^2^Immunization Safety Office, National Center for Emerging and Zoonotic Infectious Diseases, CDC; ^3^Division of Infectious Diseases, Vanderbilt University Medical Center, Nashville, Tennessee; ^4^Department of Health Research Methods, Evidence, and Impact, McMaster University, Hamilton, Ontario, Canada

## Abstract

**This report updates the 2021–22 recommendations of the Advisory Committee on Immunization Practices (ACIP) concerning the use of seasonal influenza vaccines in the United States:**

(MMWR Recomm Rep 2021;70[No. RR-5]:1–24)*. Routine annual influenza vaccination is recommended for all persons aged ≥6 months who do not have contraindications. For each recipient, a licensed and age-appropriate vaccine should be used.* *With the exception of vaccination for adults aged ≥65 years, ACIP makes no preferential recommendation for a specific vaccine when more than one licensed, recommended, and age-appropriate vaccine is available. All seasonal influenza vaccines expected to be available in the United States for the 2022–23 season are quadrivalent, containing hemagglutinin (HA) derived from one influenza A(H1N1)pdm09 virus, one influenza A(H3N2) virus, one influenza B/Victoria lineage virus, and one influenza B/Yamagata lineage virus. Inactivated influenza vaccines (IIV4s), recombinant influenza vaccine (RIV4), and live attenuated influenza vaccine (LAIV4) are expected to be available. Trivalent influenza vaccines are no longer available, but data that involve these vaccines are included for reference.*

**Influenza vaccines might be available as early as July or August, but for most persons who need only 1 dose of influenza vaccine for the season, vaccination should ideally be offered during September or October. However, vaccination should continue after October and throughout the season as long as influenza viruses are circulating and unexpired vaccine is available. For most adults (particularly adults aged ≥65 years) and for pregnant persons in the first or second trimester, vaccination during July and August should be avoided unless there is concern that vaccination later in the season might not be possible. Certain children aged 6 months through 8 years need 2 doses; these children should receive the first dose as soon as possible after vaccine is available, including during July and August. Vaccination during July and August can be considered for children of any age who need only 1 dose for the season and for pregnant persons who are in the third trimester if vaccine is available during those months:**

**Updates described in this report reflect discussions during public meetings of ACIP that were held on October 20, 2021; January 12, 2022; February 23, 2022; and June 22, 2022. Primary updates to this report include the following three topics: 1) the composition of 2022–23 U.S. seasonal influenza vaccines; 2) updates to the description of influenza vaccines expected to be available for the 2022–23 season, including one influenza vaccine labeling change that occurred after the publication of the 2021–22 ACIP influenza recommendations; and 3) updates to the recommendations concerning vaccination of adults aged ≥65 years. First, the composition of 2022–23 U.S. influenza vaccines includes updates to the influenza A(H3N2) and influenza B/Victoria lineage components. U.S.-licensed influenza vaccines will contain HA derived from an influenza A/Victoria/2570/2019 (H1N1)pdm09-like virus (for egg-based vaccines) or an influenza A/Wisconsin/588/2019 (H1N1)pdm09-like virus (for cell culture–based or recombinant vaccines); an influenza A/Darwin/9/2021 (H3N2)-like virus (for egg-based vaccines) or an influenza A/Darwin/6/2021 (H3N2)-like virus (for cell culture–based or recombinant vaccines); an influenza B/Austria/1359417/2021 (Victoria lineage)-like virus; and an influenza B/Phuket/3073/2013 (Yamagata lineage)-like virus. Second, the approved age indication for the cell culture–based inactivated influenza vaccine, Flucelvax Quadrivalent (ccIIV4), was changed in October 2021 from ≥2 years to ≥6 months. Third, recommendations for vaccination of adults aged ≥65 years have been modified. ACIP recommends that adults aged ≥65 years preferentially receive any one of the following higher dose or adjuvanted influenza vaccines: quadrivalent high-dose inactivated influenza vaccine (HD-IIV4), quadrivalent recombinant influenza vaccine (RIV4), or quadrivalent adjuvanted inactivated influenza vaccine (aIIV4). If none of these three vaccines is available at an opportunity for vaccine administration, then any other age-appropriate influenza vaccine should be used:**

**This report focuses on recommendations for the use of vaccines for the prevention and control of seasonal influenza during the 2022–23 influenza season in the United States. A brief summary of the recommendations and a link to the most recent Background Document containing additional information are available at:**

https://www.cdc.gov/vaccines/hcp/acip-recs/vacc-specific/flu.html *. These recommendations apply to U.S.-licensed influenza vaccines used according to Food and Drug Administration–licensed indications. Updates and other information are available from CDC’s influenza website (* https://www.cdc.gov/flu *). Vaccination and health care providers should check this site periodically for additional information.*

## Introduction

Influenza viruses typically circulate annually in the United States, most commonly from the late fall through the early spring. Most persons who become ill after influenza virus infection recover without serious complications or sequelae. However, influenza can be associated with serious illnesses, hospitalizations, and deaths, particularly among older adults, very young children, pregnant persons, and persons of all ages with certain chronic medical conditions (*1*–*7*). Influenza also is an important cause of missed work and school (*8*–*10*). Routine annual influenza vaccination for all persons aged ≥6 months who do not have contraindications has been recommended by CDC and the Advisory Committee on Immunization Practices (ACIP) since 2010 (*11*).

Vaccination provides important protection from influenza illness and its potential complications. The effectiveness of influenza vaccination varies depending on several factors, such as the age and health of the recipient; the type of vaccine administered; the types, subtypes (for influenza A), and lineages (for influenza B) of circulating influenza viruses; and the degree of similarity between circulating viruses and those included in the vaccine (*12*). During each of the six influenza seasons from 2010–11 through 2015–16, influenza vaccination prevented an estimated 1.6–6.7 million illnesses, 790,000–3.1 million outpatient medical visits, 39,000–87,000 hospitalizations, and 3,000–10,000 respiratory and circulatory deaths each season in the United States (*13*). During the severe 2017–18 season, notable for an unusually long duration of widespread high influenza activity throughout the United States and higher rates of outpatient visits and hospitalizations compared with recent seasons, vaccination prevented an estimated 7.1 million illnesses, 3.7 million medical visits, 109,000 hospitalizations, and 8,000 deaths (*14*), despite an overall estimated vaccine effectiveness of 38% (62% against influenza A[H1N1]pdm09 viruses, 22% against influenza A[H3N2] viruses, and 50% against influenza B viruses) (*14*).

Influenza circulated at historically low levels in the United States and globally during the 2020–21 influenza season (*15*). This was coincident with widespread implementation of nonpharmaceutical interventions (e.g., masking, social distancing, and suspension of in-person work and school) intended to prevent transmission of SARS-CoV-2 (the virus that causes COVID-19). Influenza activity increased during the 2021–22 season, although severity indicators (e.g., influenza-associated hospitalizations and deaths) were overall lower than in recent previous seasons (*16*). Timing, intensity, and severity of the 2022–23 influenza season cannot be predicted. Influenza vaccination remains an important tool for the prevention of potentially severe respiratory illness, which might decrease stress on the U.S. health care system during ongoing circulation of SARS-CoV-2. Guidance for vaccine planning during the COVID-19 pandemic is available at https://www.cdc.gov/vaccines/pandemic-guidance/index.html.

This report updates the 2021–22 ACIP recommendations regarding the use of seasonal influenza vaccines (*17*) and provides recommendations and guidance for vaccination providers regarding the use of influenza vaccines in the United States for the 2022–23 season. Various formulations of influenza vaccines are available (Table 1). Contraindications and precautions for the use of influenza vaccines are summarized (Tables 2 and 3). Abbreviations are used in this report to denote the various types of vaccines (Box).

**TABLE 1:** Influenza vaccines — United States, 2022–23 influenza season*

Trade name (manufacturer)	Presentations	Age indication	*µ*g HA (IIV4s and RIV4) or virus count (LAIV4) for each vaccine virus (per dose)	Route	Mercury (from thimerosal, if present), *µ*g/0.5 mL
**IIV4 (standard-dose, egg-based vaccines^†^)**					
Afluria Quadrivalent (Seqirus)	0.5-mL PFS^§^	≥3 yrs^§^	15 *µ*g/0.5 mL	IM^¶^	—**
	5.0-mL MDV^§^	≥6 mos^§ ^(needle and syringe) 18 through 64 yrs (jet injector)	7.5 *µ*g/0.25 mL 15 *µ*g/0.5 mL	IM^¶^	24.5
Fluarix Quadrivalent (GlaxoSmithKline)	0.5-mL PFS	≥6 mos	15 *µ*g/0.5 mL	IM^¶^	—
FluLaval Quadrivalent (GlaxoSmithKline)	0.5-mL PFS	≥6 mos	15 *µ*g/0.5 mL	IM^¶^	—
Fluzone Quadrivalent (Sanofi Pasteur)	0.5-mL PFS^††^	≥6 mos^††^	15 *µ*g/0.5 mL	IM^¶^	—
	0.5-mL SDV^††^	≥6 mos^††^	15 *µ*g/0.5 mL	IM^¶^	—
	5.0-mL MDV^††^	≥6 mos^††^	7.5 *µ*g/0.25 mL 15 *µ*g/0.5 mL	IM^¶^	25
**ccIIV4 (standard-dose, cell culture–based vaccine)**					
Flucelvax Quadrivalent (Seqirus)	0.5-mL PFS	≥6 mos	15 *µ*g/0.5 mL	IM^¶^	—
	5.0-mL MDV	≥6 mos	15 *µ*g/0.5 mL	IM^¶^	25
**HD-IIV4 (high-dose, egg-based vaccine^†^)**					
Fluzone High-Dose Quadrivalent (Sanofi Pasteur)	0.7-mL PFS	≥65 yrs	60 *µ*g/0.7 mL	IM^¶^	—
**aIIV4 (standard-dose, egg-based vaccine^†^ with MF59 adjuvant)**					
Fluad Quadrivalent (Seqirus)	0.5-mL PFS	≥65 yrs	15 *µ*g/0.5 mL	IM^¶^	—
**RIV4 (recombinant HA vaccine)**					
Flublok Quadrivalent (Sanofi Pasteur)	0.5-mL PFS	≥18 yrs	45 *µ*g/0.5 mL	IM^¶^	—
**LAIV4 (egg-based vaccine^†^)**					
FluMist Quadrivalent (AstraZeneca)	0.2-mL prefilled single-use intranasal sprayer	2 through 49 yrs	10^6.5–7.5^ fluorescent focus units/0.2 mL	NAS	—

**Abbreviations:** ACIP = Advisory Committee on Immunization Practices; FDA = Food and Drug Administration; HA = hemagglutinin; IIV4 = inactivated influenza vaccine, quadrivalent; IM = intramuscular; LAIV4 = live attenuated influenza vaccine, quadrivalent; MDV = multidose vial; NAS = intranasal; PFS = prefilled syringe; RIV4 = recombinant influenza vaccine, quadrivalent; SDV = single-dose vial.

* Vaccination providers should consult FDA-approved prescribing information for 2022–23 influenza vaccines for the most complete and updated information, including but not limited to indications, contraindications, warnings, and precautions. Package inserts for U.S.-licensed vaccines are available at https://www.fda.gov/vaccines-blood-biologics/vaccines/vaccines-licensed-use-united-states. Availability and characteristics of specific products and presentations might change or differ from what is described in this table and in the text of this report.

^† ^Although a history of severe allergic reaction (e.g., anaphylaxis) to egg is a labeled contraindication to the use of egg-based IIV4s and LAIV4, ACIP recommends that persons with a history of egg allergy may receive any licensed, recommended influenza vaccine that is otherwise appropriate for their age and health status. Those who report having had reactions to egg involving symptoms other than urticaria (e.g., angioedema or swelling, respiratory distress, lightheadedness, or recurrent emesis) or who required epinephrine or another emergency medical intervention should be vaccinated in an inpatient or outpatient medical setting (including but not necessarily limited to hospitals, clinics, health departments, and physician offices) supervised by a health care provider who is able to recognize and manage severe allergic reactions, if a vaccine other than ccIIV4 or RIV4 is used.

^§^ The approved dose volume for Afluria Quadrivalent is 0.25 mL for children aged 6 through 35 months and 0.5 mL for persons aged ≥3 years. However, 0.25-mL prefilled syringes are not expected to be available for the 2022–23 season. For children aged 6 through 35 months, a 0.25-mL dose must be obtained from a multidose vial.

^¶^ IM-administered influenza vaccines should be given by needle and syringe only, with the exception of the MDV presentation of Afluria Quadrivalent, which may alternatively be given by the PharmaJet Stratis jet injector for persons aged 18 through 64 years only. For adults and older children, the recommended site for intramuscular influenza vaccination is the deltoid muscle. The preferred site for infants and young children is the anterolateral aspect of the thigh. Additional specific guidance regarding site selection and needle length for intramuscular administration is available in the ACIP General Best Practice Guidelines for Immunization, available at https://www.cdc.gov/vaccines/hcp/acip-recs/general-recs/index.html.

** Not applicable.

^†† ^Fluzone Quadrivalent is currently approved for ages 6 through 35 months at either 0.25 mL or 0.5 mL per dose; however, 0.25-mL prefilled syringes are not expected to be available for the 2022–23 influenza season. If a prefilled syringe of Fluzone Quadrivalent is used for a child in this age group, the dose volume will be 0.5 mL per dose.

**TABLE 2:** Contraindications and precautions for the use of influenza vaccines — United States, 2022–23 influenza season*

Vaccine type	Contraindications	Precautions
Egg-based IIV4s	• History of severe allergic reaction (e.g., anaphylaxis) to any component of the vaccine^†^ or to a previous dose of any influenza vaccine (i.e., any egg-based IIV, ccIIV, RIV, or LAIV)^§^	• Moderate or severe acute illness with or without fever • History of Guillain-Barré syndrome within 6 weeks of receipt of influenza vaccine
ccIIV4	• History of severe allergic reaction (e.g., anaphylaxis) to a previous dose of any ccIIV or any component of ccIIV4^§^	• Moderate or severe acute illness with or without fever • History of Guillain-Barré syndrome within 6 weeks of receipt of influenza vaccine • History of severe allergic reaction to a previous dose of any other influenza vaccine (i.e., any egg-based IIV, RIV, or LAIV)^¶^
RIV4	• History of severe allergic reaction (e.g., anaphylaxis) to a previous dose of any RIV or any component of RIV4^§^	• Moderate or severe acute illness with or without fever • History of Guillain-Barré syndrome within 6 weeks of receipt of influenza vaccine • History of severe allergic reaction to a previous dose of any other influenza vaccine (i.e., any egg-based IIV, ccIIV, or LAIV)^¶^
LAIV4	• History of severe allergic reaction (e.g., anaphylaxis) to any component of the vaccine^†^ or to a previous dose of any influenza vaccine (i.e., any egg-based IIV, ccIIV, RIV, or LAIV)^§^ • Concomitant aspirin- or salicylate-containing therapy in children and adolescents^§^ • Children aged 2 through 4 years who have received a diagnosis of asthma or whose parents or caregivers report that a health care provider has told them during the preceding 12 months that their child had wheezing or asthma or whose medical record indicates a wheezing episode has occurred during the preceding 12 months • Children and adults who are immunocompromised due to any cause, including but not limited to immunosuppression caused by medications, congenital or acquired immunodeficiency states, HIV infection, anatomic asplenia, or functional asplenia (e.g., due to sickle cell anemia) • Close contacts and caregivers of severely immunosuppressed persons who require a protected environment • Pregnancy • Persons with active communication between the CSF and the oropharynx, nasopharynx, nose, or ear or any other cranial CSF leak • Persons with cochlear implants** • Receipt of influenza antiviral medication within the previous 48 hours for oseltamivir and zanamivir, previous 5 days for peramivir, and previous 17 days for baloxavir^††^	• Moderate or severe acute illness with or without fever • History of Guillain-Barré syndrome within 6 weeks of receipt of influenza vaccine • Asthma in persons aged ≥5 years • Other underlying medical conditions that might predispose to complications after wild-type influenza infection (e.g., chronic pulmonary, cardiovascular [except isolated hypertension], renal, hepatic, neurologic, hematologic, or metabolic disorders [including diabetes mellitus])

**Abbreviations:** ACIP = Advisory Committee on Immunization Practices; ccIIV = cell culture–based inactivated influenza vaccine (any valency); ccIIV4 = cell culture–based inactivated influenza vaccine, quadrivalent; CSF = cerebrospinal fluid; FDA = Food and Drug Administration; IIV = inactivated influenza vaccine (any valency); IIV4 = inactivated influenza vaccine, quadrivalent; LAIV = live attenuated influenza vaccine (any valency); LAIV4 = live attenuated influenza vaccine, quadrivalent; RIV = recombinant influenza vaccine (any valency); RIV4 = recombinant influenza vaccine, quadrivalent.

* When a contraindication is present, a vaccine should not be administered. When a precaution is present, vaccination should generally be deferred but might be indicated if the benefit of protection from the vaccine outweighs the risk for an adverse reaction (see ACIP General Best Practice Guidelines for Immunization, available at https://www.cdc.gov/vaccines/hcp/acip-recs/general-recs/index.html). Vaccination providers should check FDA-approved prescribing information for 2022–23 influenza vaccines for the most complete and updated information, including (but not limited to) indications, contraindications, warnings, and precautions. Package inserts for U.S.-licensed vaccines are available at https://www.fda.gov/vaccines-blood-biologics/vaccines/vaccines-licensed-use-united-states.

^†^ Although a history of severe allergic reaction (e.g., anaphylaxis) to egg is a labeled contraindication to the use of egg-based IIV4s and LAIV4, ACIP recommends that persons with a history of egg allergy may receive any licensed, recommended influenza vaccine that is otherwise appropriate for their age and health status. Those who report having had reactions to egg involving symptoms other than urticaria (e.g., angioedema or swelling, respiratory distress, lightheadedness, or recurrent emesis) or who required epinephrine or another emergency medical intervention should be vaccinated in an inpatient or outpatient medical setting, including but not necessarily limited to hospitals, clinics, health departments, and physician offices, if a vaccine other than ccIIV4 or RIV4 is used. Vaccine administration should be supervised by a health care provider who is able to recognize and manage severe allergic reactions.

^§^ Labeled contraindication noted in package insert.

^¶^ If administered, vaccination should occur in a medical setting and should be supervised by a health care provider who can recognize and manage severe allergic reactions. Providers can consider consultation with an allergist in such cases, to assist in identification of the component responsible for the allergic reaction.

** Age-appropriate injectable vaccines are recommended for persons with cochlear implant due to the potential for CSF leak, which might exist for a period after implantation. Providers might consider consultation with a specialist concerning risk for persistent CSF leak if an age-appropriate inactivated or recombinant vaccine cannot be used.

^††^ Use of LAIV4 in context of influenza antivirals has not been studied; however, interference with activity of LAIV4 is biologically plausible, and this possibility is noted in the package insert for LAIV4. In the absence of data supporting an adequate minimum interval between influenza antiviral use and LAIV4 administration, the intervals provided are based on the half-life of each antiviral. The interval between influenza antiviral receipt and LAIV4 for which interference might potentially occur might be further prolonged in the presence of medical conditions that delay medication clearance (e.g., renal insufficiency). Influenza antivirals might also interfere with LAIV4 if initiated within 2 weeks after vaccination. Persons who receive antivirals during the period starting with the specified time before receipt of LAIV4 through 2 weeks after receipt of LAIV4 should be revaccinated with an age-appropriate IIV or RIV4.

**TABLE 3:** Influenza vaccine contraindications and precautions for persons with a history of severe allergic reaction to a previous dose of an influenza vaccine* — United States, 2022–23 influenza season

Vaccine (of any valency) associated with previous severe allergic reaction (e.g., anaphylaxis)	Available 2022–23 influenza vaccines		
	Egg-based IIV4s and LAIV4	ccIIV4	RIV4
Any egg-based IIV or LAIV	Contraindication^†^	Precaution^§^	Precaution^§^
Any ccIIV	Contraindication^†^	Contraindication^†^	Precaution^§^
Any RIV	Contraindication^†^	Precaution^§^	Contraindication^†^
Unknown influenza vaccine	Allergist consultation recommended		

**Abbreviations:** ACIP = Advisory Committee on Immunization Practices; ccIIV = cell culture–based inactivated influenza vaccine (any valency); ccIIV4 = cell culture–based inactivated influenza vaccine, quadrivalent; FDA = Food and Drug Administration; IIV = inactivated influenza vaccine (any valency); IIV4 = inactivated influenza vaccine, quadrivalent; LAIV = live attenuated influenza vaccine (any valency); LAIV4 = live attenuated influenza vaccine, quadrivalent; RIV = recombinant influenza vaccine (any valency); RIV4 = recombinant influenza vaccine, quadrivalent.

* Vaccination providers should check FDA-approved prescribing information for 2022–23 influenza vaccines for the most complete and updated information, including (but not limited to) indications, contraindications, warnings, and precautions. Package inserts for U.S.-licensed vaccines are available at https://www.fda.gov/vaccines-blood-biologics/vaccines/vaccines-licensed-use-united-states.

^† ^When a contraindication is present, a vaccine should not be administered, consistent with ACIP General Best Practice Guidelines for Immunization (Kroger A, Bahta L, Hunter P. General best practice guidelines for immunization: best practices guidance of the Advisory Committee on Immunization Practices [ACIP]. https://www.cdc.gov/vaccines/hcp/acip-recs/general-recs/index.html). In addition to the contraindications based on history of severe allergic reaction to influenza vaccines that are noted in the table, each individual influenza vaccine is contraindicated for persons who have had a severe allergic reaction (e.g., anaphylaxis) to any component of that vaccine. Vaccine components can be found in package inserts. Although a history of severe allergic reaction (e.g., anaphylaxis) to egg is a labeled contraindication to the use of egg-based IIV4s and LAIV4, ACIP recommends that persons with a history of egg allergy may receive any licensed, recommended influenza vaccine that is otherwise appropriate for their age and health status. Those who report having had reactions to egg involving symptoms other than urticaria (e.g., angioedema or swelling, respiratory distress, lightheadedness, or recurrent emesis) or who required epinephrine or another emergency medical intervention should be vaccinated in an inpatient or outpatient medical setting (including but not necessarily limited to hospitals, clinics, health departments, and physician offices), if a vaccine other than ccIIV4 or RIV4 is used. Vaccine administration should be supervised by a health care provider who is able to recognize and manage severe allergic reactions.

^§ ^When a precaution is present, vaccination should generally be deferred but might be indicated if the benefit of protection from the vaccine outweighs the risk for an adverse reaction, consistent with ACIP General Best Practice Guidelines for Immunization (Kroger A, Bahta L, Hunter P. General best practice guidelines for immunization: best practices guidance of the Advisory Committee on Immunization Practices [ACIP]. https://www.cdc.gov/vaccines/hcp/acip-recs/general-recs/index.html). Providers can consider using the following vaccines in these instances; however, vaccination should occur in an inpatient or outpatient medical setting with supervision by a health care provider who is able to recognize and manage severe allergic reactions: 1) for persons with a history of severe allergic reaction (e.g., anaphylaxis) to any egg-based IIV or LAIV of any valency, the provider can consider administering ccIIV4 or RIV4; 2) for persons with a history of severe allergic reaction (e.g., anaphylaxis) to any ccIIV of any valency, the provider can consider administering RIV4; and 3) for persons with a history of severe allergic reaction (e.g., anaphylaxis) to any RIV of any valency, the provider can consider administering ccIIV4. Providers can also consider consulting with an allergist to help determine which vaccine component is responsible for the allergic reaction.

BOX **Abbreviation conventions for influenza vaccines discussed in this report**

- Main influenza vaccine types:
  **º IIV** = inactivated influenza vaccine
  **º RIV** = recombinant influenza vaccine
  **º LAIV** = live attenuated influenza vaccine
- Numerals following letter abbreviations indicate valency (the number of influenza virus hemagglutinin [HA] antigens represented in the vaccine):
  **º 4** for quadrivalent vaccines: one A(H1N1), one A(H3N2), and two B viruses (one from each lineage)
  **º 3** for trivalent vaccines: one A(H1N1), one A(H3N2), and one B virus (from one lineage)
- All influenza vaccines expected to be available in the United States for the 2022–23 season are quadrivalent vaccines. However, abbreviations for trivalent vaccines (e.g., IIV3) might be used in this report when discussing information specific to trivalent vaccines.
- Abbreviations for general vaccine categories (e.g., IIV) might be used when discussing information that is not specific to either trivalent or quadrivalent vaccines.
- Prefixes are used when necessary to refer to certain specific IIVs:
  **º a** for MF59-adjuvanted inactivated influenza vaccine (e.g., aIIV3 and aIIV4)
  **º cc** for cell culture–based inactivated influenza vaccine (e.g., ccIIV3 and ccIIV4)
  **º HD** for high-dose inactivated influenza vaccine (e.g., HD-IIV3 and HD-IIV4)
  **º SD** for standard-dose inactivated influenza vaccine (e.g. SD-IIV3 and SD-IIV4)

This report focuses on recommendations and guidance for the use of seasonal influenza vaccines for the prevention and control of influenza during the 2022–23 season in the United States. A summary of these recommendations and a Background Document containing additional information on influenza, influenza-associated illness, and influenza vaccines are available at https://www.cdc.gov/vaccines/hcp/acip-recs/vacc-specific/flu.html.

## Methods

ACIP provides annual recommendations for the use of influenza vaccines for the prevention and control of seasonal influenza in the United States. The ACIP Influenza Work Group meets by teleconference once to twice per month throughout the year. Work group membership includes several voting members of ACIP, representatives of ACIP liaison organizations, and consultants. Discussions include topics such as influenza surveillance, vaccine effectiveness and safety, vaccination coverage, program feasibility, cost effectiveness, and vaccine supply. Presentations are requested from invited experts, and published and unpublished data are discussed.

The Background Document that supplements this report is updated periodically to reflect recent additions to the literature related to recommendations made in previous seasons and minor changes in guidance for the use of influenza vaccines (e.g., guidance for timing of vaccination and other programmatic issues, guidance for dosage in specific populations, guidance for selection of vaccines for specific populations that are already recommended for vaccination, and changes that reflect use that is consistent with indications and prescribing information licensed by the Food and Drug Administration [FDA]). The summary included in the Background Document for such topics is not a systematic review; it is intended to provide an overview of current literature, with updated articles being identified primarily through a broad search for English-language articles on influenza and influenza vaccines. Typically, systematic review and evaluation of evidence using the Grading of Recommendations Assessment, Development and Evaluation (GRADE) approach (*18*) are performed for new recommendations or substantial changes in the current recommendations (e.g., expansion of the recommendation for influenza vaccination to new populations not previously recommended for vaccination or potential preferential recommendations for specific vaccines). Systematic review, GRADE, and the ACIP Evidence to Recommendations Framework were used in the development of the updated recommendations for influenza vaccination of adults aged ≥65 years discussed in this document.

Primary updates and changes to the recommendations described in this report include 1) the vaccine virus composition for 2022–23 U.S. seasonal influenza vaccines; 2) updates to the description of influenza vaccines expected to be available for the 2022–23 season, including one influenza vaccine labeling change that occurred after the publication of the 2021–22 ACIP influenza recommendations; and 3) updates to the recommendations concerning vaccination of adults aged ≥65 years. Information relevant to these changes includes the following:

- Recommendations for the composition of Northern Hemisphere influenza vaccines are made by the World Health Organization (WHO), which organizes a consultation, usually in February of each year. Surveillance data are reviewed, and candidate vaccine viruses are discussed. Information about the WHO meeting on February 25, 2022, for selection of the 2022–23 Northern Hemisphere vaccine viruses is available at https://www.who.int/news/item/25-02-2022-recommendations-announced-for-influenza-vaccine-composition-for-the-2022-2023-northern-hemisphere-influenza-season. Subsequently, FDA, which has regulatory authority over vaccines in the United States, convenes a meeting of its Vaccines and Related Biological Products Advisory Committee (VRBPAC). This committee considers the recommendations of WHO, reviews and discusses similar data, and makes a final decision regarding vaccine virus composition of influenza vaccines licensed and marketed in the United States. Materials from the VRBPAC discussion on March 3, 2022, during which the composition of the 2022–23 U.S. influenza vaccines was discussed, are available at https://www.fda.gov/advisory-committees/advisory-committee-calendar/vaccines-and-related-biological-products-advisory-committee-march-3-2022-meeting-announcement.
- Regarding recommendations concerning newly licensed influenza vaccines and changes to the licensed indications for existing vaccines, ACIP relies on FDA for review of safety, immunogenicity, and efficacy and effectiveness data pertaining to licensure and labeling of influenza vaccines. Vaccines that are expected to be available for the 2022–23 season are described (see Vaccines Available for the 2022–23 Season) (Table 1). Regulatory information pertinent to the change in age indication for Flucelvax Quadrivalent discussed in this report is available at https://www.fda.gov/vaccines-blood-biologics/vaccines/flucelvax-quadrivalent.
- Regarding updated recommendations for influenza vaccines for adults aged ≥65 years, a summary of the review of evidence on this topic is available at https://www.cdc.gov/vaccines/acip/recs/grade/influenza-older-adults.html. A summary of the ACIP Evidence to Recommendations Framework for this discussion is available at https://www.cdc.gov/vaccines/acip/recs/grade/influenza-older-adults-etr.html .

## Primary Changes and Updates

Routine annual influenza vaccination of all persons aged ≥6 months who do not have contraindications continues to be recommended. Primary updates in this report include the following:

1. The composition of the 2022–23 U.S. seasonal influenza vaccines includes updates to the influenza A(H3N2) and influenza B/Victoria components. For the 2022–23 season, U.S.-licensed influenza vaccines will contain hemagglutinin (HA) derived from an influenza A/Victoria/2570/2019 (H1N1)pdm09-like virus (for egg-based vaccines) or an influenza A/Wisconsin/588/2019 (H1N1)pdm09-like virus (for cell culture–based and recombinant vaccines); an influenza A/Darwin/9/2021 (H3N2)-like virus (for egg-based vaccines) or an influenza A/Darwin/6/2021 (H3N2)-like virus (for cell culture–based or recombinant vaccines); an influenza B/Austria/1359417/2021 (Victoria lineage)-like virus; and an influenza B/Phuket/3073/2013 (Yamagata lineage)-like virus.
2. Influenza vaccines expected to be available for the 2022–23 season, their age indications, and their presentations are described (Table 1). Afluria Quadrivalent is not expected to be available in a 0.25-mL prefilled syringe presentation. When using Afluria Quadrivalent for children aged 6 through 35 months (who require a 0.25-mL dose), the dose must be obtained from a multidose vial. One labeling change is described. In October 2021, FDA granted approval for the use of Flucelvax Quadrivalent (cell culture–based quadrivalent inactivated influenza vaccine [ccIIV4]) for children aged 6 months through <2 years. As of March 2021, Flucelvax Quadrivalent had been approved for persons aged ≥2 years. Approval for children aged 6 months through <2 years was based on results of a randomized, observer-blind study that compared immunogenicity and safety of Flucelvax Quadrivalent with that of a licensed comparator IIV4 among 2,402 children aged 6 through 47 months, of whom 894 were aged 6 through 23 months. Flucelvax Quadrivalent is now approved for persons aged ≥6 months.
3. On the basis of review of evidence concerning high-dose inactivated influenza vaccine (HD-IIV), recombinant influenza vaccine (RIV), and MF59-adjuvanted inactivated influenza vaccine (aIIV), recommendations for influenza vaccination of persons aged ≥65 years have been modified. ACIP recommends that adults aged ≥65 years preferentially receive any one of the following higher dose or adjuvanted influenza vaccines: quadrivalent high-dose inactivated influenza vaccine (HD-IIV4), quadrivalent recombinant influenza vaccine (RIV4), or quadrivalent adjuvanted inactivated influenza vaccine (aIIV4). If none of these three vaccines is available at an opportunity for vaccine administration, then any other age-appropriate influenza vaccine should be used. Higher dose vaccines include HD-IIV4 and RIV4, both of which contain a higher dose of HA antigen per virus than standard-dose vaccines (60 *µ*g for HD-IIV4 and 45 *µ*g for RIV4, compared with 15 *µ*g for standard-dose inactivated vaccines). Adjuvanted inactivated influenza vaccine (aIIV4) contains MF59 adjuvant.

## Recommendations for the Use of Influenza Vaccines, 2022–23

### Groups Recommended for Vaccination

Routine annual influenza vaccination is recommended for all persons aged ≥6 months who do not have contraindications. Recommendations regarding timing of vaccination, considerations for specific populations, the use of specific vaccines, and contraindications and precautions are summarized in the sections that follow.

### Timing of Vaccination

Because timing of the onset, peak, and decline of influenza activity varies, the ideal time to start vaccinating cannot be predicted each season. Decisions about timing necessitate balancing considerations regarding this unpredictability of the influenza season, possible waning of vaccine-induced immunity over the course of a season, and programmatic considerations. Influenza vaccines might be available as early as July or August; however, vaccination during these months is not recommended for most groups because of the possible waning of immunity over the course of the influenza season (*19**–**32*). For most persons who need only 1 dose of influenza vaccine for the season, vaccination should ideally be offered during September or October. However, vaccination should continue after October and throughout the influenza season as long as influenza viruses are circulating and unexpired vaccine is available. 

Considerations for timing of vaccination include the following:

- **For most adults (particularly adults aged ≥65 years) and for pregnant persons in the first or second trimester:** Vaccination during July and August should be avoided unless there is concern that vaccination later in the season might not be possible.
- **Children who require 2 doses:** Certain children aged 6 months through 8 years require 2 doses of influenza vaccine for the season (see Children Aged 6 Months Through 8 Years: Number of Influenza Vaccine Doses) (Figure). These children should receive their first dose as soon as possible (including during July and August, if vaccine is available) to allow the second dose (which must be administered ≥4 weeks later) to be received, ideally, by the end of October.

**FIGURE:**
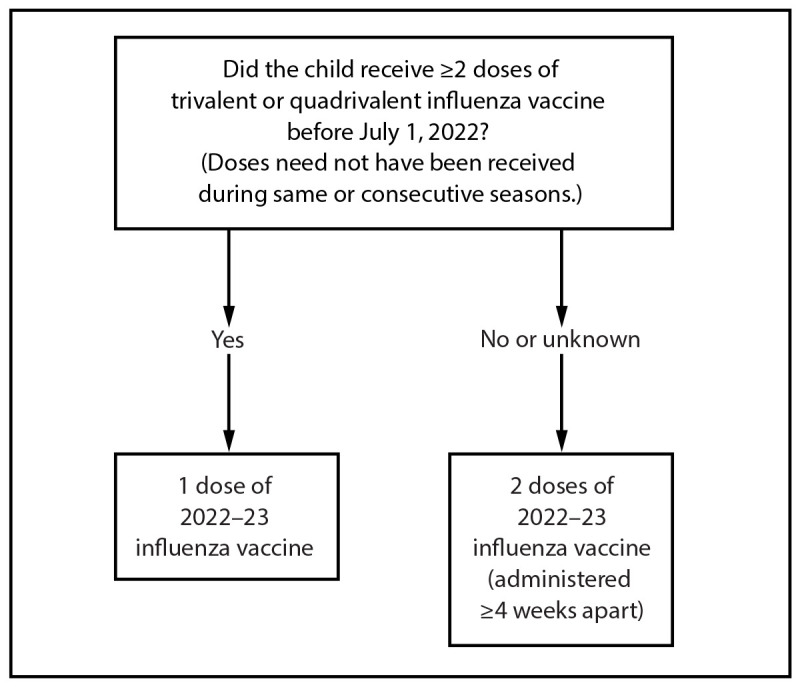
Influenza vaccine dosing algorithm for children aged 6 months through 8 years* — Advisory Committee on Immunization Practices, United States, 2022–23 influenza season * Children aged 6 months through 8 years who require 2 doses of influenza vaccine should receive their first dose as soon as possible (including during July and August, if vaccine is available) to allow the second dose (which must be administered ≥4 weeks later) to be received, ideally, by the end of October. For children aged 8 years who require 2 doses of vaccine, both doses should be administered even if the child turns age 9 years between receipt of dose 1 and dose 2.
- **Children who require only 1 dose:** Vaccination during July and August can be considered for children of any age who need only 1 dose of influenza vaccine for the season. While waning of immunity after vaccination over the course of the season has been observed among all age groups (*19**–**32*), there are fewer published studies reporting results specifically among children (*19**–**22*). Moreover, children in this group might visit health care providers during the late summer months for medical examinations before the start of school. Vaccination can be considered at this time because it represents a vaccination opportunity.
- **Pregnant persons in the third trimester:** Vaccination during July and August can be considered for pregnant persons who are in the third trimester because vaccination might reduce risk for influenza illness in their infants during the first months after birth, when they are too young to receive influenza vaccine (*33**–**36*). For pregnant persons in the first or second trimester during July and August, waiting to vaccinate until September or October is preferable, unless there is concern that later vaccination might not be possible.

Community vaccination programs should balance maximizing the likelihood of persistence of vaccine-induced protection through the season with avoiding missed opportunities to vaccinate or vaccinating after onset of influenza circulation occurs. Efforts should be structured to optimize vaccination coverage before influenza activity in the community begins. Vaccination should continue to be offered as long as influenza viruses are circulating and unexpired vaccine is available. To avoid missed opportunities for vaccination, providers should offer vaccination during routine health care visits and hospitalizations. No recommendation is made for revaccination (i.e., providing a booster dose) later in the season of persons who have been fully vaccinated for the season, regardless of when the current season vaccine was received.

During the 2022–23 influenza season, SARS-CoV-2 is expected to continue to circulate in the United States, and COVID-19 vaccinations are expected to continue. Current guidance for the administration of COVID-19 vaccines (“Coadministration of COVID-19 vaccines with other vaccines” at https://www.cdc.gov/vaccines/covid-19/clinical-considerations/interim-considerations-us.html#recommendations) indicates that these vaccines can be administered with influenza vaccines; providers should consult this page for updated information. Guidance for vaccine planning during the COVID-19 pandemic is available at https://www.cdc.gov/vaccines/pandemic-guidance/index.html.

Optimally, vaccination should occur before onset of influenza activity in the community. However, because timing of the onset, peak, and decline of influenza activity varies, the ideal time to start vaccinating cannot be predicted each season. Moreover, more than one outbreak might occur in a community in a single year. In the United States, localized outbreaks indicating the start of seasonal influenza activity can occur as early as October. However, in 29 (76%) of 38 influenza seasons from 1982–83 through 2019–20, peak influenza activity (which often is close to the midpoint of influenza activity for the season) has not occurred until January or later, and in 23 (61%) seasons, the peak was in February or later (*37*). Activity peaked in February in 17 (45%) of these seasons (*37*).

An increasing number of observational studies (*19**–**32*) have reported decreases in vaccine effectiveness with increasing time postvaccination within a single influenza season. Waning effects have not been observed consistently across age groups, influenza viruses (types, subtypes, and lineages), or seasons. Certain studies suggest waning occurs to a greater degree against influenza A(H3N2) viruses than against influenza A(H1N1) or influenza B viruses (*26**,**27*). This effect also might vary with recipient age; in certain studies, waning was more pronounced among older adults (*19**,**23**,**26**,**30**,**32*) and younger children (*19*). Relatively fewer reports include results specific to children (*19**–**22*); findings suggestive of waning have been reported in certain studies (*19**–**21*) but not others (*22*). Rates of decline in vaccine effectiveness also varied. A multiseason (2011–12 through 2014–15) analysis from the U.S. Influenza Vaccine Effectiveness (U.S. Flu VE) Network found that vaccine effectiveness decreased by approximately 7% per month for influenza A(H3N2) and influenza B and 6%–11% per month for influenza A(H1N1)pdm09 (*25*). Vaccine effectiveness remained greater than zero for at least 5–6 months after vaccination. In the Hospitalized Adult Influenza Vaccine Effectiveness Network (HAIVEN) during the 2015–16 through 2018–19 seasons, vaccine effectiveness against influenza-associated hospitalizations declined by approximately 8%–9% per month for all adults and approximately 10%–11% per month for those aged ≥65 years (*32*). An analysis of the 2010–11 through 2013–14 seasons noted estimated effectiveness ranging from 54% to 67% during days 0–180 postvaccination; estimated vaccine effectiveness was not significant during the period between days 181 and 365 (*20*). A third multiseason analysis (2010–11 through 2014–15) conducted in Europe noted a decline in vaccine effectiveness to 0% at 111 days postvaccination against influenza A(H3N2) viruses. Vaccine effectiveness against influenza B viruses decreased more slowly, and vaccine effectiveness against influenza A(H1N1)pdm09 viruses remained roughly stable at 50%–55% throughout the influenza season (*27*). A meta-analysis of 14 studies examining waning of influenza vaccine effectiveness using the test-negative design found a significant decline in effectiveness within 180 days after vaccination against influenza A (H3N2) and influenza B but not against influenza A(H1N1) (*38*). In addition to the factors observed to be associated with waning immunity across studies, observed decreases in protection might be at least in part attributable to bias, unmeasured confounding, or the late-season emergence of antigenic drift variants of influenza viruses that are less well-matched to the vaccine viruses.

Varying data concerning the presence and rate of waning immunity after influenza vaccination, coupled with the unpredictable timing of the influenza season each year, prevent determination of an optimal time to vaccinate. Programmatic issues also are a consideration. Although delaying vaccination might result in greater immunity later in the season, deferral also might result in missed opportunities to vaccinate as well as difficulties in vaccinating a population within a more constrained period. The potential contributions of these factors among persons aged ≥65 years have been assessed using a simulated mathematical model examining various scenarios of vaccination timing, timing of onset of the influenza season, vaccine effectiveness, and rate of waning (*39*). In this model, during an influenza season beginning in October and peaking in January, delaying vaccination until October resulted in more hospitalizations if >14% of persons aged ≥65 years who would have been vaccinated in August or September failed to get vaccinated. However, these predictions varied considerably with assumed timing of season onset, rate of waning immunity, and vaccine effectiveness.

Vaccination efforts should continue throughout the season because the duration of the influenza season varies, and influenza activity might not occur in certain communities until February, March, or later. Providers should offer influenza vaccine routinely, and organized vaccination campaigns should continue throughout the influenza season, including after influenza activity has begun in the community. Although vaccination by the end of October is recommended, vaccine administered in December or later, even if influenza activity has already begun, might be beneficial in most influenza seasons. Providers should offer influenza vaccination to unvaccinated persons who have already become ill with influenza during the season because the vaccine might protect them against other circulating influenza viruses.

### Guidance for Influenza Vaccination in Specific Populations and Situations

#### Populations at Higher Risk for Medical Complications Attributable to Severe Influenza

All persons aged ≥6 months who do not have contraindications should be vaccinated annually. However, vaccination to prevent influenza is particularly important for persons who are at increased risk for severe illness and complications from influenza and for influenza-related outpatient, emergency department, or hospital visits. When vaccine supply is limited, vaccination efforts should focus on vaccination of persons at higher risk for medical complications attributable to severe influenza who do not have contraindications. These persons include the following (no hierarchy is implied by order of listing):

- All children aged 6 through 59 months.
- All persons aged ≥50 years.
- Adults and children who have chronic pulmonary (including asthma), cardiovascular (excluding isolated hypertension), renal, hepatic, neurologic, hematologic, or metabolic disorders (including diabetes mellitus).
- Persons who are immunocompromised due to any cause (including but not limited to immunosuppression caused by medications or HIV infection).
- Persons who are or will be pregnant during the influenza season.
- Children and adolescents (aged 6 months through 18 years) who are receiving aspirin- or salicylate-containing medications and who might be at risk for experiencing Reye syndrome after influenza virus infection.
- Residents of nursing homes and other long-term care facilities.
- American Indian or Alaska Native persons.
- Persons who are extremely obese (body mass index ≥40 for adults).

An IIV4 or RIV4 (as appropriate for the recipient’s age) is suitable for persons in all risk groups. LAIV4 is not recommended for certain populations, including certain of these listed groups. Contraindications and precautions for the use of LAIV4 are noted (Tables 2 and 3).

#### Persons Who Live with or Care for Persons at Higher Risk for Influenza-Related Complications

All persons aged ≥6 months without contraindications should be vaccinated annually. However, emphasis also should be placed on vaccination of persons who live with or care for those who are at increased risk for medical complications attributable to severe influenza. When vaccine supply is limited, vaccination efforts should focus on administering vaccination to persons at higher risk for influenza-related complications as well as persons who live with or care for such persons, including the following:

- Health care personnel, including all paid and unpaid persons working in health care settings who have the potential for exposure to patients or to infectious materials. These personnel might include but are not limited to physicians, nurses, nursing assistants, nurse practitioners, physician assistants, therapists, technicians, emergency medical service personnel, dental personnel, pharmacists, laboratory personnel, autopsy personnel, students and trainees, contractual staff persons, and others not directly involved in patient care but who might be exposed to infectious agents (e.g., clerical, dietary, housekeeping, laundry, security, maintenance, administrative, and billing staff persons and volunteers). ACIP guidance for vaccination of health care personnel has been published previously (*40*).
- Household contacts (including children aged ≥6 months) and caregivers of children aged ≤59 months (<5 years) and adults aged ≥50 years, particularly contacts of children aged <6 months.
- Household contacts (including children aged ≥6 months) and caregivers of persons with medical conditions that put them at higher risk for severe complications from influenza.

Health care personnel and persons who are contacts of persons in these groups (with the exception of contacts of severely immunocompromised persons who require a protected environment) may receive any influenza vaccine that is otherwise indicated. Persons who care for severely immunocompromised persons requiring a protected environment should not receive LAIV4. ACIP and the Healthcare Infection Control Practices Advisory Committee (HICPAC) have previously recommended that health care personnel who receive LAIV should avoid providing care for severely immunocompromised persons requiring a protected environment for 7 days after vaccination and that hospital visitors who have received LAIV should avoid contact with such persons for 7 days after vaccination (*41*). However, such persons need not be restricted from caring for or visiting less severely immunocompromised persons.

#### Influenza Vaccination of Persons with COVID-19

Vaccination of persons who have tested positive for COVID-19 or who are in quarantine after an exposure should include multiple considerations, such as whether bringing the person into a vaccination setting could expose others to COVID-19, whether the person is acutely ill and the severity of the illness, the presence of risk factors for severe influenza illness, the likelihood of being able to vaccinate at a later date, and the desire to avoid confusing postvaccination symptoms with those of COVID-19. Usually, persons who are in quarantine or isolation should not be brought to a vaccination setting if doing so could expose others to COVID-19. For those who have moderate or severe COVID-19, vaccination should usually be deferred until they have recovered, which is consistent with ACIP General Best Practice Guidelines for Immunization (*42*). For persons who have mild or asymptomatic COVID-19, further deferral might be considered to avoid confusing COVID-19 symptoms with postvaccination reactions. Because recommendations for vaccination of this population might continue to evolve, clinicians should check CDC guidance (https://www.cdc.gov/vaccines/pandemic-guidance/index.html) for up-to-date information.

#### Children Aged 6 Through 35 Months: Influenza Vaccine Dose Volumes 

Five IIV4s are approved for children aged ≥6 months (Table 1). Four of these vaccines are egg based (Afluria Quadrivalent, Fluarix Quadrivalent, Flulaval Quadrivalent, and Fluzone Quadrivalent), and one is cell culture based (Flucelvax Quadrivalent). For these vaccines, the approved dose volumes for children aged 6 through 35 months are as follows (Table 4):

- Afluria Quadrivalent: 0.25 mL per dose. However, 0.25-mL prefilled syringes are not expected to be available for the 2022–23 season. For children aged 6 through 35 months, a 0.25-mL dose must be obtained from a multidose vial (*43*).
- Fluarix Quadrivalent: 0.5 mL per dose.
- Flucelvax Quadrivalent: 0.5 mL per dose.
- FluLaval Quadrivalent: 0.5 mL per dose.
- Fluzone Quadrivalent: either 0.25 mL or 0.5 mL per dose. Per the package insert, each dose may be given at either volume (*44*); however, the 0.25-mL prefilled syringes are no longer available.

For all of these IIV4s, persons aged ≥36 months (≥3 years) should receive 0.5 mL per dose.

Alternatively, healthy children aged ≥24 months (≥2 years) may receive LAIV4, 0.2 mL intranasally (0.1 mL in each nostril). LAIV4 is not recommended for certain populations and is not approved for children aged <2 years (see Contraindications and Precautions for the Use of LAIV4) (Table 2). RIV4 is not approved for children aged <18 years. High-dose inactivated influenza vaccine (HD-IIV4) and adjuvanted inactivated influenza vaccine (aIIV4) are not approved for persons aged <65 years.

Care should be taken to administer an age-appropriate vaccine at the appropriate volume for each dose. For IIV4s, the recommended volume may be administered from a prefilled syringe containing the appropriate volume (as supplied by the manufacturer), a single-dose vial, or a multidose vial. Single-dose vials should be used for only 1 dose, and multidose vials should be used only for the maximum number of doses specified in the package insert. Any vaccine remaining in a vial after the maximum number of doses has been removed should be discarded, regardless of the volume of the doses obtained or any remaining volume in the vial.

#### Children Aged 6 Months Through 8 Years: Number of Influenza Vaccine Doses 

Children aged 6 months through 8 years require 2 doses of influenza vaccine administered a minimum of 4 weeks apart during their first season of vaccination for optimal protection (*45**–**48*). Determination of the number of doses needed is based on 1) the child’s age at the time of the first dose of 2022–23 influenza vaccine and 2) the number of doses of influenza vaccine received in previous influenza seasons.

- For children aged 6 months through 8 years, the number of doses of influenza vaccine needed for the 2022–23 influenza season is determined as follows (Figure):
  º Those who have previously received ≥2 total doses of trivalent or quadrivalent influenza vaccine ≥4 weeks apart before July 1, 2022, require only 1 dose for the 2022–23 season. The 2 previous doses of influenza vaccine do not need to have been received in the same season or consecutive seasons.
  º Those who have not previously received ≥2 doses of trivalent or quadrivalent influenza vaccine ≥4 weeks apart before July 1, 2022, or whose previous influenza vaccination history is unknown, require 2 doses for the 2022–23 season. The interval between the 2 doses should be ≥4 weeks. Children aged 6 months through 8 years who require 2 doses of influenza vaccine should receive their first dose as soon as possible (including during July and August, if vaccine is available) to allow the second dose (which must be administered ≥4 weeks later) to be received, ideally, by the end of October. Two doses are recommended even if the child turns age 9 years between receipt of dose 1 and dose 2.
- Adults and children aged ≥9 years need only 1 dose of influenza vaccine for the 2022–23 season.

#### Pregnant Persons

Pregnant and postpartum persons have been observed to be at higher risk for severe illness and complications from influenza, particularly during the second and third trimesters. Influenza vaccination during pregnancy is associated with reduced risk for respiratory illness and influenza among pregnant and postpartum persons as well as infants during the first several months of life (*33**–**36**,**49*). ACIP and the American College of Obstetricians and Gynecologists recommend that persons who are pregnant or who might be pregnant or postpartum during the influenza season receive influenza vaccine (*50*). Any licensed, recommended, and age-appropriate IIV4 or RIV4 may be used. LAIV4 should not be used during pregnancy but can be used postpartum. Influenza vaccine can be administered at any time during pregnancy (i.e., during any trimester), before and during the influenza season. Early vaccination (i.e., during July and August) can be considered for persons who are in the third trimester during these months if vaccine is available because this can provide protection for the infant during the first months of life when they are too young to be vaccinated.

Although experience with the use of IIVs during pregnancy is substantial, data specifically reflecting administration of influenza vaccines during the first trimester are limited (see Safety of Influenza Vaccines in the supplementary Background Document). Most studies have not noted an association between influenza vaccination and adverse pregnancy outcomes, including spontaneous abortion (miscarriage) (*51**–**61*). One observational Vaccine Safety Datalink (VSD) study conducted during the 2010–11 and 2011–12 seasons noted an association between receipt of IIV containing influenza A(H1N1)pdm09 and risk for miscarriage in the 28 days after receipt of IIV, when an H1N1pdm09-containing vaccine also had been received the previous season (*62*). However, in a larger VSD follow-up study, IIV was not associated with an increased risk for miscarriage during the 2012–13, 2013–14, and 2014–15 seasons, regardless of previous season vaccination (*63*).

Substantially less experience exists with more recently licensed IIVs (e.g., quadrivalent and cell culture–based vaccines) during pregnancy than with previously available products. For RIV (available as RIV3 from 2013–14 through 2017–18 and as RIV4 since 2017–18), data are limited to reports of pregnancies occurring incidentally during clinical trials, Vaccine Adverse Event Reporting System (VAERS) reports, and pregnancy registries. Pregnancy registries and surveillance studies exist for certain products, for which information can be found in package inserts.

#### Older Adults

ACIP recommends that adults aged ≥65 years preferentially receive any one of the following higher dose or adjuvanted influenza vaccines: quadrivalent high-dose inactivated influenza vaccine (HD-IIV4), quadrivalent recombinant influenza vaccine (RIV4), or quadrivalent adjuvanted inactivated influenza vaccine (aIIV4). If none of these three vaccines is available at an opportunity for vaccine administration, then any other age-appropriate influenza vaccine should be administered.

Older adults (aged ≥65 years) are at increased risk for severe influenza-associated illness, hospitalization, and death compared with younger persons (*4**,**16**,**64*). Influenza vaccines are often less effective in this population (*65*). HD-IIV, RIV, and aIIV have been evaluated in comparison with nonadjuvanted SD-IIVs in this age group. Two of these vaccines, HD-IIV and RIV, are higher dose vaccines, which contain an increased dose of HA antigen per virus compared with nonadjuvanted SD-IIVs (60 *µ*g for HD-IIV4 and 45 *µ*g for RIV4, compared with 15 *µ*g for standard-dose inactivated vaccines). The adjuvanted vaccine contains 15 *µ*g of HA per virus, similarly to nonadjuvanted SD-IIVs, but contains the adjuvant MF59.

HD-IIV, RIV, and aIIV have shown relative benefit compared with SD-IIVs in certain studies, with the most evidence available for HD-IIV3. Randomized efficacy studies comparing these vaccines with nonadjuvanted SD-IIVs against laboratory-confirmed influenza outcomes are few in number (*66**–**68*) and cover few influenza seasons. Observational studies, predominantly retrospective cohort studies using diagnostic code–defined (rather than laboratory-confirmed) outcomes, are more numerous and include more influenza seasons (*69**–**79*). Certain observational studies have reported relative benefit for HD-IIV, RIV, and aIIV in comparison with nonadjuvanted SD-IIVs, particularly in prevention of influenza-associated hospitalizations. The size of this relative benefit has varied from season to season and is not seen in all studies in all seasons, making it difficult to generalize the findings to all or most seasons. Studies directly comparing HD-IIV, RIV, and aIIV with one another are few and do not support a conclusion that any one of these vaccines is consistently superior to the others across seasons (*70**–**72**,**79**–**81*).

Of note, for the 2020–21 season, quadrivalent formulations of high-dose (HD-IIV4) and adjuvanted (aIIV4) influenza vaccines were introduced. Trivalent formulations of these vaccines are no longer available. Data summarizing comparisons of these newer quadrivalent formulations relative to nonadjuvanted SD-IIV4s against laboratory-confirmed influenza outcomes are not yet available.

Literature concerning the efficacy, effectiveness, and safety of HD-IIV, RIV, and aIIV versus nonadjuvanted SD-IIVs and of each of these three vaccines with one another was reviewed, focusing on published studies performed during nonpandemic influenza seasons. A description of the systematic review and GRADE is available at https://www.cdc.gov/vaccines/acip/recs/grade/influenza-older-adults.html. An abbreviated summary follows.

**Randomized Studies Comparing HD-IIV, RIV, and aIIV with Nonadjuvanted SD-IIVs: Prevention of Influenza Illnesses.** Randomized studies comparing HD-IIV, RIV, and aIIV with nonadjuvanted SD-IIVs against laboratory-confirmed influenza illness are few in number and were conducted over few influenza seasons. HD-IIV3 was more effective than SD-IIV3 in prevention of polymerase chain reaction (PCR)- or culture-confirmed influenza-like illness (ILI) in a two-season randomized study conducted among 32,000 persons aged ≥65 years (relative efficacy: 24%; 95% CI: 10%–36%; certainty level: 1, high) (*66*). Two single-season randomized trials of RIV versus nonadjuvanted SD-IIV, one a comparison of RIV3 versus nonadjuvanted SD-IIV3 that assessed culture-confirmed ILI (*67*) and the other a comparison of RIV4 versus nonadjuvanted SD-IIV4 that examined PCR-confirmed ILI (*68*), did not demonstrate relative benefit of RIV among those aged ≥65 years (pooled relative efficacy: 18%; 95% CI: −17% to 43%; certainty level: 2, moderate). The larger of these two studies noted a relative benefit of RIV4 over nonadjuvanted SD-IIV4 in prevention of PCR-confirmed influenza among the full study population of persons aged ≥50 years (relative efficacy: 30%; 95% CI: 10%–47%) as well as against culture-confirmed ILI among those aged ≥65 years (relative efficacy: 42%; 95% CI: 9%–65%) (*68*). No data are available from randomized trials of aIIV versus nonadjuvanted SD-IIVs against laboratory-confirmed influenza outcomes during nonpandemic influenza seasons.

**Randomized Studies Comparing HD-IIV, RIV, and aIIV with Nonadjuvanted SD-IIVs: Prevention of Influenza-Associated Hospitalizations and Other Serious Events.** No data are available from randomized trials evaluating prevention of laboratory-confirmed influenza-associated hospitalizations as a primary outcome. In a secondary analysis from a two-season randomized trial of HD-IIV3 versus nonadjuvanted SD-IIV3 assessing serious adverse events (SAEs) (including hospitalizations) associated with laboratory confirmation of influenza performed outside of the study (*82*) and a post hoc analysis of pneumonia- and influenza-related hospitalizations from a randomized study of HD-IIV3 versus nonadjuvanted SD-IIV4 (*83*), there was no difference in risk for these events between the two groups (certainty level: 2, moderate). However, additional data are available from two single-season, cluster-randomized studies conducted among U.S. nursing homes (in which nursing homes were randomized to vaccine groups rather than individual persons) that examined prevention of pneumonia and influenza diagnostic–coded hospitalizations. One such study noted a benefit of HD-IIV3 relative to nonadjuvanted SD-IIV3 (adjusted relative risk: 0.79; 95% CI: 0.66–0.95; certainty level: 2, moderate) (*84*). The second noted a benefit of aIIV3 relative to nonadjuvanted SD-IIV3 (adjusted hazard ratio: 0.79; 95% CI: 0.65–0.96; certainty level: 2, moderate) (*85*).

**Observational Studies Comparing HD-IIV, RIV, and aIIV with Nonadjuvanted SD-IIVs: Prevention of Influenza-Associated Hospitalizations and Deaths.** Observational studies comparing HD-IIV and aIIV with nonadjuvanted SD-IIVs are more numerous than randomized studies and cover more influenza seasons. Many of these studies assessed diagnostic code–defined (rather than laboratory-confirmed) outcomes. In these studies, an overall modest relative benefit in prevention of diagnostic code–defined influenza-associated hospitalizations has been observed for HD-IIV3 (*70**–**77*) and aIIV3 (*70**–**72*) versus nonadjuvanted SD-IIV3s. Relative benefit was not found in every study for all evaluated seasons for either HD-IIV3 or aIIV3. Published observational studies of RIV are fewer than for HD-IIV and aIIV. A retrospective analysis of relative effectiveness of RIV4 versus SD-IIV4 against influenza-coded hospitalizations among Medicare beneficiaries during the 2019–20 season noted a relative effectiveness of 17% (95% CI: 9%–24%; certainty level: 3, low) (*72*). Observational studies that address relative benefit in protection against influenza-associated deaths are limited. Two retrospective cohort studies including three influenza seasons noted a relative benefit of HD-IIV3 compared with nonadjuvanted SD-IIV3 (*69**,**78*) diagnostic code–defined deaths (pooled rate ratio: 0.69; 95% CI: 0.57–0.84; certainty level: 3, low).

**Observational Studies Comparing Effectiveness of HD-IIV, RIV, and aIIV with One Another. **Data reflecting comparisons of HD-IIV, aIIV, and RIV with one another are more limited than comparisons with nonadjuvanted SD-IIVs. Observational studies have compared HD-IIV3 versus aIIV3 (*70**–**72**,**79**–**81*), HD-IIV3 versus RIV4 (*72*), and aIIV3 versus RIV4 (*72*). A retrospective cohort analysis noted relative effectiveness of RIV4 compared with HD-IIV3 (relative effectiveness: 11%; 95% CI: 3%–18%; certainty level: 3, low) and with aIIV3 (relative effectiveness: 11%; 95% CI: 3%–17%; certainty level: 3, low); these data covered only a single influenza season. Data do not point to a consistent relative benefit of one of these three influenza vaccines over another across multiple seasons.

**Safety.** In comparative safety studies, certain injection site and systemic reactions were observed more frequently in older persons vaccinated with HD-IIV3 and aIIV3 compared with nonadjuvanted SD-IIV3 (*86**,**87*). In a randomized trial of RIV4 versus nonadjuvanted SD-IIV4 among persons aged ≥50 years, frequencies of solicited injection site events were similar or lower among RIV4 recipients; frequency of fever was similar between the two vaccines. Frequencies of SAEs were similar between the two groups, and none was judged to be related to a study vaccine (*68*). One postlicensure randomized clinical trial in the United States evaluated the comparative safety of aIIV3 compared with HD-IIV3 in 757 adults aged ≥65 years (*88*). For the primary outcome, the proportion of participants who reported moderate to severe injection site pain that limited or prevented activity after aIIV3 (12 participants [3.2%]) was noninferior compared with the proportion reporting this outcome after vaccination with HD-IIV3 (22 participants [5.8%]). No participant sought medical care for a solicited reaction symptom, and none had a SAE determined by study investigators to be related to vaccine within 43 days after vaccination. 

#### Immunocompromised Persons

ACIP recommends that persons with compromised immunity (including but not limited to persons with congenital and acquired immunodeficiency states, persons who are immunocompromised due to medications, and persons with anatomic and functional asplenia) should receive an age-appropriate IIV4 or RIV4. ACIP recommends that LAIV4 not be used for these groups because of the uncertain but biologically plausible risk for disease attributable to the live vaccine virus. Use of LAIV4 in persons with these and other conditions is discussed in more detail (see Dosage, Administration, Contraindications, and Precautions) (Table 2).

Immunocompromised states comprise a heterogeneous range of conditions with varying risks for severe infections. In many instances, limited data are available regarding the effectiveness of influenza vaccines in the setting of specific immunocompromised states (*89*). Timing of vaccination might be a consideration (e.g., vaccinating during a period either before or after an immunocompromising intervention). The Infectious Diseases Society of America has published detailed guidance for the selection and timing of vaccines for persons with specific immunocompromising conditions (*90*). Immune response to influenza vaccines might be blunted in persons with certain conditions, such as congenital immune deficiencies, and in persons receiving cancer chemotherapy or immunosuppressive medications.

#### Persons with a History of Guillain-Barré Syndrome After Influenza Vaccination

A history of Guillain-Barré syndrome (GBS) within 6 weeks of a previous dose of any type of influenza vaccine is considered a precaution for influenza vaccination (Table 2). Persons who are not at higher risk for severe influenza complications (see Populations at Higher Risk for Medical Complications Attributable to Severe Influenza) and who are known to have experienced GBS within 6 weeks of a previous influenza vaccination typically should not be vaccinated. As an alternative to vaccination, providers might consider using influenza antiviral chemoprophylaxis for these persons (*91*). However, the benefits of influenza vaccination might outweigh the possible risks for certain persons who have a history of GBS within 6 weeks after receipt of influenza vaccine and who also are at higher risk for severe complications from influenza.

#### Persons with a History of Egg Allergy

Most available influenza vaccines, with the exceptions of RIV4 (Flublok Quadrivalent, licensed for those aged ≥18 years) and ccIIV4 (Flucelvax Quadrivalent, licensed for those aged ≥6 months), are prepared by propagation of virus in embryonated eggs and might contain trace amounts of egg proteins, such as ovalbumin. For persons who report a history of egg allergy, ACIP recommends the following:

- Persons with a history of egg allergy who have experienced only urticaria (hives) after exposure to egg should receive influenza vaccine. Any licensed, recommended influenza vaccine (i.e., any IIV4, RIV4, or LAIV4) that is otherwise appropriate for the recipient’s age and health status can be used.
- Persons who report having had reactions to egg involving symptoms other than urticaria (e.g., angioedema or swelling, respiratory distress, lightheadedness, or recurrent vomiting) or who required epinephrine or another emergency medical intervention can similarly receive any licensed, recommended influenza vaccine (i.e., any IIV4, RIV4, or LAIV4) that is otherwise appropriate for their age and health status. If a vaccine other than ccIIV4 or RIV4 is used, the selected vaccine should be administered in an inpatient or outpatient medical setting, including but not necessarily limited to hospitals, clinics, health departments, and physician offices. Vaccine administration should be supervised by a health care provider who is able to recognize and manage severe allergic reactions.

All vaccination providers should be familiar with their office emergency plan and be certified in cardiopulmonary resuscitation (*42*). No postvaccination observation period is recommended specifically for egg-allergic persons. However, ACIP recommends that vaccination providers consider observing patients (seated or supine) for 15 minutes after administration of any vaccine to decrease the risk for injury should syncope occur (*42*).

#### Persons with Previous Allergic Reactions to Influenza Vaccines

As is the case for all vaccines, influenza vaccines contain various components that might cause allergic and anaphylactic reactions. Most influenza vaccine package inserts list among contraindications to their use a history of previous severe allergic reaction (e.g., anaphylaxis) to any component of the vaccine or to a previous dose of any influenza vaccine. For ccIIV4 and RIV4, a history of a severe allergic reaction to any vaccine component is listed as a contraindication; no labeled contraindication is specified for a history of allergic reaction to any other influenza vaccine. However, severe allergic reactions, although rare, can occur after influenza vaccination, even among persons with no previous reactions or known allergies. Although vaccine components can be found in package inserts, identifying the causative component without further evaluation (i.e., through evaluation and testing for specific allergies) can be difficult. Severe allergic reactions after vaccination with an RIV have been reported to VAERS, some of which have occurred among persons reporting previous allergic reactions to egg or to influenza vaccines and which might represent a predisposition to development of allergic manifestations in affected persons (*92**–**94*). Because these rare but severe allergic reactions can occur, ACIP recommends the following for persons with a history of severe allergic reaction to a previous dose of an influenza vaccine (Table 3):

- For egg-based IIV4s and LAIV4:
  º A history of severe allergic reaction (e.g., anaphylaxis) to any influenza vaccine (i.e., any egg-based IIV, ccIIV, RIV, or LAIV of any valency) is a contraindication to future receipt of all egg-based IIV4s and LAIV4. Each individual egg-based IIV4 and LAIV4 is also contraindicated for persons who have had a severe allergic reaction (e.g., anaphylaxis) to any component of that vaccine (excluding egg) (see Persons with a History of Egg Allergy).
- For ccIIV4:
  º A history of a severe allergic reaction (e.g., anaphylaxis) to any egg-based IIV, RIV, or LAIV of any valency is a precaution for the use of ccIIV4. If ccIIV4 is administered in such instances, vaccination should occur in an inpatient or outpatient medical setting and should be supervised by a health care provider who is able to recognize and manage severe allergic reactions. Providers also can consider consultation with an allergist to help determine the vaccine component responsible for the allergic reaction.
  º A history of a severe allergic reaction (e.g., anaphylaxis) to any ccIIV of any valency or to any component of ccIIV4 is a contraindication to future receipt of ccIIV4.
- For RIV4:
  º A history of a severe allergic reaction (e.g., anaphylaxis) to any egg-based IIV, ccIIV, or LAIV of any valency is a precaution for the use of RIV4. If RIV4 is administered in such instances, vaccination should occur in an inpatient or outpatient medical setting and should be supervised by a health care provider who is able to recognize and manage severe allergic reactions. Providers can also consider consultation with an allergist to help determine the vaccine component responsible for the allergic reaction.
  º A history of a severe allergic reaction (e.g., anaphylaxis) to any RIV of any valency or to any component of RIV4 is a contraindication to future receipt of RIV4.

#### Vaccination Issues for Travelers

In temperate climate regions of the Northern and Southern Hemispheres, influenza activity is seasonal, occurring during approximately October–May in the Northern Hemisphere and April–September in the Southern Hemisphere. In the tropics, influenza might occur throughout the year. Travelers can be exposed to influenza when traveling to an area where influenza is circulating or when traveling as part of large tourist groups (e.g., on cruise ships) that include persons from areas of the world where influenza viruses are circulating (*95**–**98*).

Travelers who want to reduce their risk for influenza should consider influenza vaccination, preferably at least 2 weeks before departure. In particular, persons who live in the United States and are at higher risk for influenza complications and who were not vaccinated with influenza vaccine during the previous Northern Hemisphere fall or winter should consider receiving influenza vaccination before departure if they plan to travel to the tropics, to the Southern Hemisphere during the Southern Hemisphere influenza season (April–September), or with organized tourist groups or on cruise ships to any location. Persons at higher risk who received the previous season’s influenza vaccine before travel should consult with their health care provider to discuss the risk for influenza and other travel-related diseases before embarking on travel during the summer. All persons (regardless of risk status) who are vaccinated in preparation for travel before the upcoming influenza season’s vaccine is available should receive the current vaccine the following fall or winter.

Influenza vaccine formulated for the Southern Hemisphere might differ in viral composition from the Northern Hemisphere vaccine. For persons traveling to the Southern Hemisphere during the Southern Hemisphere influenza season, receipt of a current U.S.-licensed Southern Hemisphere influenza vaccine formulation before departure might be reasonable but might not be feasible because of limited access to or unavailability of Southern Hemisphere formulations in the United States. Most Southern Hemisphere influenza vaccine formulations are not licensed in the United States, and they are typically not commercially available. More information on influenza vaccines and travel is available at https://wwwnc.cdc.gov/travel/diseases/influenza-seasonal-zoonotic-and-pandemic.

#### Use of Influenza Antiviral Medications

Administration of IIV4 or RIV4 to persons receiving influenza antiviral medications for treatment or chemoprophylaxis of influenza is acceptable. Data concerning vaccination with LAIV4 in the setting of influenza antiviral use are not available. However, influenza antiviral medications might interfere with the action of LAIV4 because this vaccine contains live influenza viruses.

The package insert for LAIV4 notes that antiviral agents might reduce the effectiveness of the vaccine if given within the interval from 48 hours before to 14 days after vaccination (*99*). However, the newer influenza antivirals peramivir and baloxavir have longer half-lives than oseltamivir and zanamivir, approximately 20 hours for peramivir (*100*) and 79 hours for baloxavir (*101*), and could interfere with the replication of LAIV4 if administered >48 hours before vaccination. Potential interactions between influenza antivirals and LAIV4 have not been studied, and the ideal intervals between administration of these medications and LAIV4 are not known. Assuming a period of at least 5 half-lives for substantial decrease in drug levels (*102*), a reasonable assumption is that that peramivir might interfere with the mechanism of LAIV4 if administered from 5 days before through 2 weeks after vaccination and baloxavir might interfere if administered from 17 days before through 2 weeks after vaccination. The interval between influenza antiviral receipt and LAIV4 during which interference might occur could be further prolonged in the presence of medical conditions that delay medication clearance (e.g., renal insufficiency). Persons who receive these medications during these periods before or after receipt of LAIV4 should be revaccinated with another appropriate influenza vaccine (e.g., IIV4 or RIV4).

#### Administration of Influenza Vaccines with Other Vaccines

IIV4s and RIV4 may be administered simultaneously or sequentially with other inactivated vaccines or live vaccines. Injectable vaccines that are given concomitantly should be administered at separate anatomic sites. LAIV4 can be administered simultaneously with other live or inactivated vaccines. However, if two live vaccines are not given simultaneously, at least 4 weeks should pass after administration of one live vaccine (such as LAIV4) before another live vaccine is administered (*42*).

Current guidance concerning administration of current U.S.-approved or -authorized COVID-19 vaccines indicates that these vaccines may be given with influenza vaccines (https://www.cdc.gov/vaccines/covid-19/clinical-considerations/interim-considerations-us.html). Providers should be aware of the potential for increased reactogenicity with coadministration and should consult updated CDC guidance as more information becomes available. If administered simultaneously, COVID-19 vaccines and influenza vaccines that might be more likely to cause an injection site reaction (e.g., aIIV4 or HD-IIV4) should be administered in different limbs, if possible. In an interim analysis of a study of concomitant administration of HD-IIV4 and a booster dose of an mRNA COVID-19 vaccine (administered in separate upper arm sites) compared with administration of either vaccine alone among 296 persons aged ≥65 years, overall reactogenicity up to 7 days postvaccination was similar between the coadministration group and the group that received the mRNA COVID-19 vaccine alone; reactogenicity rates in the group receiving HD-IIV4 alone were lower. No SAEs were observed. Immune response was similar between the mRNA COVID-19 and coadministration groups (*103*).

Relatively limited data are available on the concomitant administration of influenza vaccines with other vaccines. Studies of live attenuated zoster vaccine and IIV3 (*104*) or IIV4 (*105*) among persons aged ≥50 years noted similar antibody responses whether the two vaccines were administered concomitantly or 4 weeks apart. In certain studies, reduced responses have been noted to 13-valent pneumococcal conjugate vaccine (PCV13) (*106**,**107*), tetanus antigens (*108*), and pertussis antigens (*108*) when coadministered with IIV3 to adults; in most instances, the clinical significance of this is uncertain. Simultaneous administration of IIV4 and 23-valent pneumococcal polysaccharide vaccine (PPSV23) to persons aged ≥65 years was associated with lower seroprotection rates to one influenza B antigen at 4–6 weeks postvaccination compared with sequential administration 2 weeks apart. Seroprotection was not significantly different between the two groups for any of the four influenza antigens at 6 months postvaccination (*109*). Reassuring safety profiles have been noted for simultaneous administration of IIVs with live attenuated zoster vaccine (*104**,**105*); PCV13 (*106**,**107*); PPSV23 (*109**,**110*); tetanus toxoid, reduced diphtheria toxoid, and acellular pertussis (Tdap) vaccine among adults (*108*); and Tdap in pregnancy (*111*). Although increased prevalence of injection site or systemic adverse reactions has been noted with concurrent administration in certain studies, these symptoms have usually been reported to be mild or moderate.

Among children aged 6 through 23 months, coadministration of IIV3 and PCV13 was associated with increased risk for fever on the day of vaccination and the day after (i.e., days 0–1 postvaccination) in an observational study conducted during the 2011–12 season (*112*). A randomized clinical trial during the 2017–18 influenza season suggested that delaying IIV4 administration by 2 weeks in children receiving diphtheria and tetanus toxoids and acellular pertussis (DTaP) and PCV13 did not reduce fever prevalence after vaccination (*113*). Increased risk for febrile seizures in this age group has been noted within days 0–1 after coadministration of IIV with PCV7, PCV13, or DTaP vaccines during the 2006–07 through 2010–11 seasons (*114*) and with PCV13 during the 2014–15 season (*115*). Although concerning to parents, most febrile seizures are brief and have a good prognosis (*116*). After considering the risks and benefits, no changes in the recommendations for administration of these vaccines were made. Surveillance of febrile seizures is ongoing through VAERS, and the VSD annual influenza safety surveillance includes monitoring for seizures after vaccinations. Studies of concomitant administration of LAIV with other vaccines are limited. Concurrent administration of LAIV3 with measles, mumps, and rubella (MMR) and varicella vaccine to children was not associated with diminished immunogenicity of antigens in any of the vaccines in one study (*117*); diminished response to rubella was observed in another study examining coadministration of LAIV3 and MMR (*118*). No safety concerns were noted in these studies.

In recent years, several vaccines containing nonaluminum adjuvants have been licensed for use in the United States for the prevention of various infectious diseases. These include AS01B (in Shingrix, recombinant zoster subunit vaccine) (*119*), MF59 (in Fluad Quadrivalent [aIIV4]) (*120*), and cytosine phosphoguanine oligodeoxynucleotide (in Heplisav-B, a recombinant hepatitis B surface antigen vaccine) (*121*). Data are limited regarding coadministration of these vaccines with other adjuvanted or nonadjuvanted vaccines, including COVID-19 vaccines. Coadministration of Shingrix with nonadjuvanted IIV4 has been studied, and no evidence of decreased immunogenicity or safety concerns was noted (*122*). The immunogenicity and safety of simultaneous or sequential administration of two nonaluminum adjuvant–containing vaccines have not been evaluated, and the ideal interval between such vaccines when given sequentially is not known. In the study of Shingrix and IIV4 (*122*), most reactogenicity symptoms resolved within 4 days. Because of the limited data on the safety of simultaneous administration of two or more vaccines containing nonaluminum adjuvants and the availability of nonadjuvanted influenza vaccine options, selection of a nonadjuvanted influenza vaccine may be considered in situations in which influenza vaccine and another vaccine containing a nonaluminum adjuvant are to be administered concomitantly. However, influenza vaccination should not be delayed if a specific vaccine is not available. As recommended for all vaccines, vaccines with nonaluminum adjuvants should be administered at separate anatomic sites from other vaccines that are given concomitantly (*42*).

## Influenza Vaccine Composition and Available Vaccines

### Influenza Vaccine Composition for the 2022–23 Season

All influenza vaccines licensed in the United States will contain components derived from influenza viruses antigenically similar to those recommended by FDA (https://www.fda.gov/advisory-committees/advisory-committee-calendar/vaccines-and-related-biological-products-advisory-committee-march-3-2022-meeting-announcement). All influenza vaccines expected to be available in the United States for the 2022–23 season will be quadrivalent vaccines. For the 2022–23 season, U.S. egg-based influenza vaccines (i.e., vaccines other than ccIIV4 and RIV4) will contain HA derived from

- an influenza A/Victoria/2570/2019 (H1N1)pdm09-like virus,
- an influenza A/Darwin/9/2021 (H3N2)-like virus,
- an influenza B/Austria/1359417/2021 (Victoria lineage)-like virus, and
- an influenza B/Phuket/3073/2013 (Yamagata lineage)-like virus.

For the 2022–23 season, U.S. cell culture–based inactivated (ccIIV4) and recombinant (RIV4) influenza vaccines will contain HA derived from

- an influenza A/Wisconsin/588/2019 (H1N1)pdm09-like virus,
- an influenza A/Darwin/6/2021 (H3N2)-like virus,
- an influenza B/Austria/1359417/2021 (Victoria lineage)-like virus, and
- an influenza B/Phuket/3073/2013 (Yamagata lineage)-like virus.

### Vaccines Available for the 2022–23 Season

Availability of specific types and brands of licensed seasonal influenza vaccines in the United States is determined by the manufacturers of the vaccines. Information presented concerning vaccines expected to be available and their approved indications and usage reflects current knowledge and is subject to change.

Various influenza vaccines will be available for the 2022–23 season (Table 1). For many vaccine recipients, more than one type or brand of vaccine might be appropriate within approved indications and ACIP recommendations. A licensed influenza vaccine that is appropriate for the recipient’s age and health status should be used. Specific age indications for licensed influenza vaccines are summarized (Table 1). Current prescribing information should be consulted for authoritative, up-to-date information. Contraindications and precautions for the different types of influenza vaccines are summarized (Tables 2 and 3), as are dose volumes (Table 4).

**TABLE 4:** Dose volumes for inactivated influenza vaccines approved for children aged 6 through 35 months* — United States, 2022–23 influenza season

Trade name (Manufacturer)	Dose volume for children aged 6 through 35 mos (*µ*g HA per vaccine virus)
Afluria Quadrivalent (Seqirus)	0.25 mL (7.5 *µ*g)^†^
Fluarix Quadrivalent (GlaxoSmithKline)	0.5 mL (15 *µ*g)
Flucelvax Quadrivalent (Seqirus)	0.5 mL (15 *µ*g)
FluLaval Quadrivalent (GlaxoSmithKline)	0.5 mL (15 *µ*g)
Fluzone Quadrivalent (Sanofi Pasteur)	0.5 mL (15 *µ*g)^§^

**Abbreviation:** HA = hemagglutinin.

* For persons aged ≥36 months (≥3 years), the dose volume is 0.5 mL per dose for all inactivated influenza vaccines with the exception of Fluzone High-Dose Quadrivalent (HD-IIV4), which is licensed for persons aged ≥65 years and for which the dose volume is 0.7 mL per dose.

^†^ The approved dose volume for Afluria Quadrivalent is 0.25 mL for children aged 6 through 35 months and 0.5 mL for persons aged ≥3 years. However, 0.25-mL prefilled syringes are not expected to be available for the 2022–23 season. For children aged 6 through 35 months, a 0.25-mL dose must be obtained from a multidose vial.

^§^ Per the package insert, Fluzone Quadrivalent is currently approved for children aged 6 through 35 months at either 0.25 mL or 0.5 mL per dose; however, 0.25-mL prefilled syringes are no longer available. If a prefilled syringe of Fluzone Quadrivalent is used for a child in this age group, the dose volume will be 0.5 mL per dose. The 0.5-mL single-dose vials should be accessed for only 1 dose and multidose vials for only 10 doses, regardless of the volume of the doses obtained or any remaining volume in the vial. Any vaccine remaining in a vial after the maximum number of doses has been removed should be discarded.

Not all influenza vaccines are likely to be uniformly available in any specific practice setting or geographic locality. Vaccination should not be delayed to obtain a specific product when an appropriate one is available. Within these guidelines and approved indications, ACIP makes no preferential recommendation for the use of any one influenza vaccine over another when more than one licensed, recommended, and age-appropriate vaccine is available, with the exception of selection of influenza vaccines for persons aged ≥65 years (see Older Adults).

Since the publication of the previous season’s recommendations, FDA has approved a labeling change for Flucelvax Quadrivalent (see Recent Influenza Vaccine Labeling Changes). Additional new licensures and changes to FDA-approved labeling might occur after publication of this report. As these changes occur and new vaccines become available, they will be reflected in the online version of Table 1, available at https://www.cdc.gov/flu/professionals/acip/2022-2023/acip-table.htm.

### Dosage, Administration, Contraindications, and Precautions

#### Quadrivalent Inactivated Influenza Vaccines (IIV4s)

**Available Vaccines.** As in recent seasons, various inactivated influenza vaccines (IIVs) are expected to be available for 2022–23 (Table 1); all are expected to be quadrivalent (IIV4s). Standard-dose, nonadjuvanted IIV4s are licensed for persons aged as young as 6 months. However, for certain IIV4s, the approved dose volume for children aged 6 through 35 months differs from that for older children and adults (Table 4). Two IIV4s, the MF59-adjuvanted IIV4 (aIIV4) and the high-dose IIV4 (HD-IIV4), are approved for persons aged ≥65 years. Care should be taken to administer the appropriate dose volume of an age-appropriate vaccine to each recipient.

Standard-dose, nonadjuvanted IIV4s contain 15 *μ*g of HA per vaccine virus in a 0.5-mL dose (7.5 *μ*g of HA per vaccine virus in a 0.25-mL dose). For 2022–23, this category is expected to include five different vaccines (Table 1). Four of these are egg-based vaccines, and one is a cell culture–based vaccine. All are approved for persons aged ≥6 months. Egg-based and cell culture–based vaccines differ in the substrate in which reference vaccine viruses supplied to the manufacturer are propagated in quantities sufficient to produce the needed number of doses of vaccine. For the IIV4s Afluria Quadrivalent (*43*), Fluarix Quadrivalent (*123*), FluLaval Quadrivalent (*124*), and Fluzone Quadrivalent (*44*), reference vaccine viruses are propagated in eggs. For Flucelvax Quadrivalent, reference vaccine viruses are propagated in Madin-Darby canine kidney cells instead of eggs (*125*).

Two additional IIV4s that will be available for the 2022–23 season are approved for persons aged ≥65 years. These vaccines are egg based. Quadrivalent high-dose inactivated influenza vaccine (Fluzone High-Dose Quadrivalent; HD-IIV4) contains 60 *μ*g of HA per vaccine virus (240 *μ*g total) in a 0.7-mL dose (*126*). Quadrivalent adjuvanted inactivated influenza vaccine (Fluad Quadrivalent; aIIV4) contains 15 *μ*g of HA per vaccine virus (60 *μ*g total) and MF59 adjuvant (*120*).

**Dosage and Administration.** Standard-dose nonadjuvanted IIV4s are approved for children aged as young as 6 months. Certain of these IIV4s are approved at different dose volumes for very young children than for older children and adults. Care should be taken to administer an age-appropriate vaccine at the approved dose volume for each needed dose (see Children Aged 6 Through 35 Months: Influenza Vaccine Dose Volumes) (Tables 1 and 4):

- Afluria Quadrivalent: The approved dose volume for children aged 6 through 35 months is 0.25 mL per dose. Persons aged ≥36 months (≥3 years) should receive 0.5 mL per dose.
- Fluarix Quadrivalent: The approved dose volume is 0.5 mL per dose for all persons aged ≥6 months.
- Flucelvax Quadrivalent: The approved dose volume is 0.5 mL per dose for all persons aged ≥6 months.
- FluLaval Quadrivalent: The approved dose volume is 0.5 mL per dose for all persons aged ≥6 months.
- Fluzone Quadrivalent: The approved dose volume for children aged 6 through 35 months is either 0.25 mL or 0.5 mL per dose. Persons aged ≥36 months (≥3 years) should receive 0.5 mL per dose.

If prefilled syringes are not available, the appropriate volume can be administered from a single-dose or multidose vial. If a 0.5-mL single-dose vial is used for a 0.25-mL dose for a child aged 6 through 35 months, only half of the vial volume should be administered, and the remaining half should be discarded. Of note, dose volume is distinct from the number of doses. Children in this age group who require 2 doses for 2022–23 need 2 separate doses administered ≥4 weeks apart, regardless of the specific IIV4 used and volume given for each dose (see Children Aged 6 Months Through 8 Years: Number of Influenza Vaccine Doses) (Figure).

For children aged 36 months (3 years) through 17 years and adults aged ≥18 years, the dose volume for IIV4s is 0.5 mL per dose, with the exception of Fluzone High-Dose Quadrivalent (HD-IIV4, licensed for persons aged ≥65 years), for which the correct volume is 0.7 mL per dose. If a smaller vaccine dose (e.g., 0.25 mL) is inadvertently administered to a person aged ≥36 months, the remaining volume needed to make a full dose should be administered during the same vaccination visit or, if measuring the needed remaining volume is a challenge, administering a repeat dose at the full volume is acceptable. If the error is discovered later (after the recipient has left the vaccination setting), a full dose should be administered as soon as the recipient can return. Vaccination with a formulation approved for adult use should be counted as a single dose if inadvertently administered to a child.

IIV4s are administered intramuscularly (IM). For adults and older children, the deltoid muscle is the preferred site. Infants and younger children should be vaccinated in the anterolateral thigh. Additional specific guidance regarding site selection and needle length for IM injection is provided in the ACIP General Best Practice Guidelines for Immunization (*42*).

One IIV4, Afluria Quadrivalent, is licensed for IM injection via the PharmaJet Stratis jet injector for persons aged 18 through 64 years (*43*). Persons in this age group may receive Afluria Quadrivalent via either needle and syringe or this specific jet injection device. Children aged 6 months through 17 years and adults aged ≥65 years should receive this vaccine by needle and syringe only. No other IIV4s are licensed for administration by jet injector.

**Contraindications and Precautions for the Use of IIV4s.** Manufacturer package inserts and updated CDC and ACIP guidance should be consulted for information on contraindications and precautions for individual influenza vaccines. Each IIV, whether egg-based or cell culture–based, has a labeled contraindication for persons with a history of a severe allergic reaction to any component of that vaccine (Table 2). Although egg is a component of all IIV4s other than ccIIV4, ACIP makes specific recommendations for the use of influenza vaccine for persons with egg allergy (see Persons with a History of Egg Allergy). All egg-based IIV4s are contraindicated in persons who have had a severe allergic reaction (e.g., anaphylaxis) to a previous dose of any influenza vaccine (any egg-based IIV, ccIIV, RIV, or LAIV of any valency). Use of ccIIV4 is contraindicated in persons who have had a severe allergic reaction (e.g., anaphylaxis) to any ccIIV of any valency. A history of severe allergic reaction (e.g., anaphylaxis) to any other influenza vaccine (i.e., any egg-based IIV, RIV, or LAIV of any valency) is a precaution for the use of ccIIV4 (see Persons with Previous Allergic Reactions to Influenza Vaccines) (Tables 2 and 3). If ccIIV4 is administered in such an instance, vaccination should occur in an inpatient or outpatient medical setting and should be supervised by a health care provider who is able to recognize and manage severe allergic reactions. Providers can also consider consultation with an allergist to help identify the vaccine component responsible for the reaction. Information about vaccine components can be found in the package inserts for each vaccine. Prophylactic use of antiviral agents is an option that can be considered for preventing influenza among persons who cannot receive vaccine, particularly for those who are at higher risk for medical complications attributable to severe influenza (*91*).

Moderate or severe acute illness with or without fever is a general precaution for vaccination (*42*). A history of GBS within 6 weeks after receipt of a previous dose of influenza vaccine is considered a precaution for the use of all influenza vaccines (Table 2).

#### Quadrivalent Recombinant Influenza Vaccine (RIV4)

**Available Vaccine.** One recombinant influenza vaccine, Flublok Quadrivalent (RIV4), is expected to be available during the 2022–23 influenza season. RIV4 is approved for persons aged ≥18 years. This vaccine contains recombinant HA produced in an insect cell line using genetic sequences from cell-derived influenza viruses and is manufactured without the use of influenza viruses or eggs (*127*).

**Dosage and Administration:** RIV4 is administered by IM injection via needle and syringe. A 0.5-mL dose contains 45 *μ*g of HA derived from each vaccine virus (180 *μ*g total).

**Contraindications and Precautions for the Use of RIV4.** RIV4 is contraindicated in persons who have had a severe allergic reaction (e.g., anaphylaxis) to a previous dose of any RIV of any valency or any component of RIV4. A history of a severe allergic reaction (e.g., anaphylaxis) to any other influenza vaccine (i.e., any egg-based IIV, ccIIV, or LAIV of any valency) is a precaution for the use of RIV4. If RIV4 is administered in such an instance, vaccination should occur in an inpatient or outpatient medical setting and should be supervised by a health care provider who is able to recognize and manage severe allergic reactions. Providers can also consider consulting with an allergist to help identify the vaccine component responsible for the reaction. Moderate or severe acute illness with or without fever is a general precaution for vaccination (*42*). A history of GBS within 6 weeks after receipt of a previous dose of influenza vaccine is considered a precaution for the use of all influenza vaccines (Table 2). RIV4 is not licensed for children aged <18 years.

#### Quadrivalent Live Attenuated Influenza Vaccine (LAIV4)

**Available Vaccine.** One live attenuated influenza vaccine, FluMist Quadrivalent (LAIV4), is expected to be available during the 2022–23 influenza season. LAIV4 is approved for persons aged 2 through 49 years. LAIV4 contains live attenuated influenza viruses that are propagated in eggs. These viruses are cold adapted (so that they replicate efficiently at 25°C) and temperature sensitive (so that their replication is restricted at higher temperatures, 39°C for influenza A viruses and 37°C for influenza B viruses). These viruses replicate in the nasopharynx, which is necessary to promote an immune response (*99*). No preference is expressed for LAIV4 versus other influenza vaccines used within specified indications.

**Dosage and Administration.** LAIV4 is administered intranasally using the supplied prefilled, single-use sprayer containing 0.2 mL of vaccine. Approximately 0.1 mL (i.e., half of the total sprayer contents) is sprayed into the first nostril while the recipient is in the upright position. An attached dose-divider clip is removed from the sprayer to permit administration of the second half of the dose into the other nostril. If the recipient sneezes immediately after administration, the dose should not be repeated. However, if nasal congestion is present that might impede delivery of the vaccine to the nasopharyngeal mucosa, deferral of administration should be considered until resolution of the illness, or another appropriate vaccine should be administered instead. Each total dose of 0.2 mL contains 10^6.5–7.5^ fluorescent focus units of each vaccine virus (*99*).

**Contraindications and Precautions for the Use of LAIV4.** Conditions considered by ACIP to be contraindications and precautions for the use of LAIV4 are summarized (Table 2). These include two labeled contraindications that appear in the package insert (*99*) and other conditions for which there is uncertain but biologically plausible potential risk associated with live viruses or limited data for use of LAIV.

Contraindications to use of LAIV4 include the following:

- Severe allergic reaction (e.g., anaphylaxis) to any component of the vaccine or to a previous dose of any influenza vaccine (i.e., any egg-based IIV, ccIIV, RIV, or LAIV of any valency; a labeled contraindication noted in the package insert). However, ACIP makes an exception for allergy to egg (see Persons with a History of Egg Allergy).
- Children and adolescents receiving concomitant aspirin- or salicylate-containing medications, because of the potential risk for Reye syndrome (a labeled contraindication noted in the package insert).
- Children aged 2 through 4 years who have received a diagnosis of asthma or whose parents or caregivers report that a health care provider has told them during the preceding 12 months that their child had wheezing or asthma or whose medical record indicates a wheezing episode has occurred during the preceding 12 months.
- Children and adults who are immunocompromised due to any cause, including but not limited to immunosuppression caused by medications, congenital or acquired immunodeficiency states, HIV infection, anatomic asplenia, or functional asplenia (such as that due to sickle cell anemia).
- Close contacts and caregivers of severely immunosuppressed persons who require a protected environment.
- Pregnancy.
- Persons with active communication between the cerebrospinal fluid (CSF) and the oropharynx, nasopharynx, nose, or ear or any other cranial CSF leak.
- Persons with cochlear implants, because of the potential for CSF leak that might exist for a period after implantation (providers might consider consultation with a specialist concerning the risk for persistent CSF leak if an age-appropriate inactivated or recombinant vaccine cannot be used).
- Receipt of influenza antiviral medication within the previous 48 hours for oseltamivir and zanamivir, previous 5 days for peramivir, and previous 17 days for baloxavir. The interval between influenza antiviral receipt and LAIV4 during which interference might potentially occur might be further prolonged in the presence of medical conditions that delay medication clearance (e.g., renal insufficiency).

Precautions for use of LAIV4 include the following:

- Moderate or severe acute illness with or without fever.
- History of GBS within 6 weeks after receipt of any influenza vaccine.
- Asthma in persons aged ≥5 years.
- Other underlying medical condition (other than those listed under contraindications) that might predispose to complications after wild-type influenza virus infection (e.g., chronic pulmonary, cardiovascular [except isolated hypertension], renal, hepatic, neurologic, hematologic, or metabolic disorders [including diabetes mellitus]).

### Recent Influenza Vaccine Labeling Changes

#### Flucelvax Quadrivalent

Since the publication of the 2021–22 ACIP influenza vaccine recommendations, a labeling change for Flucelvax Quadrivalent (ccIIV4) has occurred. Flucelvax Quadrivalent was initially approved in 2016 for persons aged ≥4 years. Approval for persons aged ≥18 years was based on a randomized immunogenicity and safety trial that compared Flucelvax Quadrivalent with the previously approved trivalent formulation of Flucelvax (ccIIV3), which had previously been licensed for persons aged ≥18 years on the basis of data from a randomized clinical efficacy trial. Approval for children aged 4 through 17 years also was based on immunogenicity and safety data compared with ccIIV3, with a postmarketing requirement to conduct a clinical efficacy study (*128*). In March 2021, FDA approved Flucelvax Quadrivalent for persons aged ≥2 years on the basis of a randomized clinical efficacy trial conducted among 4,514 children aged ≥2 to <18 years over three influenza seasons (Southern Hemisphere 2017 and Northern Hemisphere 2017–18 and 2018–19) (*129*).

Subsequently, in October 2021, FDA approved Flucelvax Quadrivalent for persons aged ≥6 months. Approval was based on a randomized immunogenicity and safety study conducted among 2,402 children aged 6 through 47 months (of whom 894 were aged 6 through 23 months). Children were randomized in a 2:1 ratio to receive either Flucelvax Quadrivalent (0.5 mL/dose containing 15 *µ*g HA per virus for all ages) or a licensed comparator egg-based IIV4 (0.25 mL/dose containing 7.5 *µ*g HA per virus for those aged 6 through 35 months and 0.5 mL/dose containing 15 *µ*g HA per virus for those aged 36 through 47 months) (*130*). Flucelvax Quadrivalent met prespecified immunogenicity criteria for all four viruses. No new safety signals were noted, and prevalence of solicited injection site and systemic reactions was similar between the two groups.

## Storage and Handling of Influenza Vaccines

In all instances, approved manufacturer packaging information should be consulted for authoritative guidance concerning storage and handling of specific influenza vaccines. Typically, influenza vaccines should be protected from light and stored at temperatures that are recommended in the package insert. Recommended storage temperatures are typically 36°F–46°F (2°C–8°C) and should be maintained at all times with adequate refrigeration and temperature monitoring. Vaccine that has frozen should be discarded. Specific recommendations for appropriate refrigerators and temperature monitoring equipment can be found in the Vaccine Storage and Handling Toolkit, available at https://www.cdc.gov/vaccines/hcp/admin/storage/toolkit/index.html.

Vaccines should not be used beyond the expiration date on the label. In addition to the expiration date, multidose vials also might have a beyond-use date (BUD), which specifies the number of days the vaccine can be kept once first accessed. After being accessed for the first dose, multidose vials should not be used after the BUD. If no BUD is provided, then the listed expiration date is to be used. Multidose vials should be returned to recommended storage conditions between uses. Package information might also specify a maximum number of doses contained in multidose vials (regardless of remaining volume). No more than the specified number of doses should be removed, and any remainder should be discarded. Single-dose vials should not be accessed for more than 1 dose. Providers should contact the manufacturer for information on permissible temperature excursions and other departures from recommended storage and handling conditions that are not discussed in the package labeling.

## Additional Sources of Information Regarding Influenza and Influenza Vaccines

### Influenza Surveillance, Prevention, and Control

Updated information regarding influenza surveillance, detection, prevention, and control is available at https://www.cdc.gov/flu. U.S. surveillance data are updated weekly throughout the year on FluView (https://www.cdc.gov/flu/weekly) and can be viewed in FluView Interactive (https://www.cdc.gov/flu/weekly/fluviewinteractive.htm). In addition, periodic updates regarding influenza are published in *MMWR* (https://www.cdc.gov/mmwr/index.html). Additional information regarding influenza and influenza vaccines can be obtained from CDCINFO by calling 1-800-232-4636. State and local health departments should be consulted about availability of influenza vaccines, access to vaccination programs, information related to state or local influenza activity, reporting of influenza outbreaks and influenza-related pediatric deaths, and advice concerning outbreak control.

### Vaccine Adverse Event Reporting System

The National Childhood Vaccine Injury Act of 1986 requires health care providers to report any adverse event listed by the vaccine manufacturer as a contraindication to future doses of the vaccine or any adverse event listed in the VAERS Table of Reportable Events Following Vaccination (https://vaers.hhs.gov/docs/VAERS_Table_of_Reportable_Events_Following_Vaccination.pdf) that occurs within the specified period after vaccination. In addition to mandated reporting, health care providers are encouraged to report any clinically significant adverse event after vaccination to VAERS. Information on how to report a vaccine adverse event is available at https://vaers.hhs.gov/index.html.

### National Vaccine Injury Compensation Program

The National Vaccine Injury Compensation Program (VICP), established by the National Childhood Vaccine Injury Act of 1986, as amended, is a mechanism through which compensation can be provided to persons who might have been injured as a result of receiving a vaccine covered by VICP. The Vaccine Injury Table (https://www.hrsa.gov/sites/default/files/hrsa/vicp/vaccine-injury-table-01-03-2022.pdf) lists the vaccines covered by VICP and the associated injuries and conditions that might receive a legal presumption of causation. If the injury or condition is not in the table or does not meet the requirements in the table, persons must prove that the vaccine caused the injury or condition. Claims must be filed with specified time frames. Persons of all ages who receive a VICP-covered vaccine might be eligible to file a claim. Additional information is available at https://www.hrsa.gov/vaccine-compensation or by calling 1-800-338-2382.

### Additional Resources

#### ACIP Statements

- General Best Practice Guidelines for Immunization: Best Practices Guidance of the Advisory Committee on Immunization Practices (ACIP): https://www.cdc.gov/vaccines/hcp/acip-recs/general-recs/index.html
- Immunization of Health Care Personnel: Recommendations of the Advisory Committee on Immunization Practices (ACIP), 2011. MMWR Recomm Rep 2011;60(No. RR-7):1–45: https://www.cdc.gov/mmwr/preview/mmwrhtml/rr6007a1.htm
- Recommended Adult Immunization Schedule for Ages 19 Years or Older, United States: https://www.cdc.gov/vaccines/schedules/hcp/imz/adult.html
- Recommended Child and Adolescent Immunization Schedule for Ages 18 Years or Younger, United States: https://www.cdc.gov/vaccines/schedules/hcp/imz/child-adolescent.html

#### COVID-19 Vaccine Recommendations and Guidance

- ACIP recommendations for the use of COVID-19 vaccines: https://www.cdc.gov/vaccines/hcp/acip-recs/vacc-specific/covid-19.html
- Clinical Care Considerations for COVID-19 Vaccination (contains clinical guidance and links to current ACIP recommendations): https://www.cdc.gov/vaccines/covid-19/clinical-considerations/index.html
- Interim Clinical Considerations for Use of COVID-19 Vaccines Currently Approved or Authorized in the United States: https://www.cdc.gov/vaccines/covid-19/clinical-considerations/covid-19-vaccines-us.html
- FDA COVID-19 Vaccines page: https://www.fda.gov/emergency-preparedness-and-response/coronavirus-disease-2019-covid-19/covid-19-vaccines

#### Vaccine Information Sheets

- IIV4 and RIV4: https://www.cdc.gov/vaccines/hcp/vis/vis-statements/flu.pdf
- LAIV4: https://www.cdc.gov/vaccines/hcp/vis/vis-statements/flulive.pdf

#### Influenza Vaccine Package Inserts

- https://www.fda.gov/vaccines-blood-biologics/vaccines/vaccines-licensed-use-united-states

#### CDC Influenza Antiviral Guidance

- Influenza Antiviral Medications: Summary for Clinicians: https://www.cdc.gov/flu/professionals/antivirals/summary-clinicians.htm

#### Infectious Diseases Society of America Influenza Antiviral Guidance

- Clinical Practice Guidelines by the Infectious Diseases Society of America: 2018 Update on Diagnosis, Treatment, Chemoprophylaxis, and Institutional Outbreak Management of Seasonal Influenza: https://academic.oup.com/cid/article/68/6/e1/5251935

#### American Academy of Pediatrics Guidance

- American Academy of Pediatrics Recommendations for Prevention and Control of Influenza in Children (Red Book Online): https://redbook.solutions.aap.org/selfserve/ sspage.aspx?selfservecontentid=influenza-resources

#### Infectious Diseases Society of America Guidance for Vaccination of Immunocompromised Hosts

- 2013 IDSA Clinical Practice Guideline for Vaccination of the Immunocompromised Host: https://academic.oup.com/cid/article/58/3/e44/336537

#### American College of Obstetricians and Gynecologists

- Influenza Vaccination During Pregnancy, ACOG Committee Opinion No. 732: https://www.acog.org/clinical/clinical-guidance/committee-opinion/articles/2018/04/influenza-vaccination-during-pregnancy
